# Development of the Validated Stability-Indicating Method for the Determination of Vortioxetine in Bulk and Pharmaceutical Formulation by HPLC-DAD, Stress Degradation Kinetics Studies and Detection of Degradation Products by LC-ESI-QTOF-MS

**DOI:** 10.3390/molecules27061883

**Published:** 2022-03-14

**Authors:** Karol Wróblewski, Małgorzata Szultka-Młyńska, Daria Janiszewska, Anna Petruczynik, Bogusław Buszewski

**Affiliations:** 1 Department of Experimental and Clinical Pharmacology, University of Rzeszów, Kopisto 2a, 35-959 Rzeszow, Poland; 2 Laboratory for Innovative Research in Pharmacology, University of Rzeszów, Kopisto 2a, 35-959 Rzeszow, Poland; 3 Interdisciplinary Center for Preclinical and Clinical Research, University of Rzeszów, Werynia 2A, 36-100 Kolbuszowa, Poland; 4 Department of Environmental Chemistry and Bioanalytics, Faculty of Chemistry, Nicolaus Copernicus University, Gagarina 7, 87-100 Torun, Poland; szultka.malgorzata@wp.pl (M.S.-M.); janiszewska_daria@doktorant.umk.pl (D.J.); bbusz@chem.umk.pl (B.B.); 5 Department of Inorganic Chemistry, Medical University of Lublin, Chodźki 4a, 20-093 Lublin, Poland; annapetruczynik@poczta.onet.pl; 6 Centre for Modern Interdisciplinary Technologies, Nicolaus Copernicus University, Wileńska 4, 87-100 Torun, Poland

**Keywords:** vortioxetine, HPLC-DAD, LC-ESI-QTOF-MS, degradation kinetic, pharmaceutical formulation, qualitative and quantitative analysis

## Abstract

Vortioxetine (VOR) is a new antidepressant drug used to treat major depressive disorder. In this work, a novel, simple, rapid, accurate, precise, selective, stability-indicating, and fully validated high-performance liquid chromatography method with diode array detection (HPLC-DAD) was developed to determine VOR in bulk and pharmaceutical formulations. A Polar-RP column was used, with a mobile phase consisting of acetonitrile (ACN), methanol (MeOH), acetate buffer pH 3.5, and addition of diethylamine (DEA) in the isocratic elution mode. Assessing the stability of the VOR is fundamental to guarantee the efficacy, safety, and quality of drug products. In this study, the VOR active pharmaceutical ingredient (API) and tablets were subjected to a detailed study of forced degradation, using several degrading agents (acid, alkaline, water, heat, light, and oxidation agents). The developed HPLC-DAD method allows the collection of all the essential data to determine degradation kinetics. It was found that the decomposition of vortioxetine is fragile towards oxidative conditions and photolysis, yielding the first-order and second-order kinetic reaction in the above stress conditions, respectively. The degradation products (DPs) were identified by the high-resolution liquid chromatography coupled with electrospray ionization-quadrupole-time of flight-mass spectrometry (LC-ESI-QTOF-MS) method. The HPLC-DAD method was successfully applied for the quantification of VOR in tablets. Additionally, in silico toxicity prediction of the DPs was performed.

## 1. Introduction

Vortioxetine, VOR (1-[2-(2,4-dimethyl-phenylsulfanyl)-phenyl]-piperazine) is a novel antidepressant used for the treatment of major depressive disorder (MDD). VOR exhibits a multimodal activity [1]. It is a 5-HT1B receptor partial agonist, 5-HT1A receptor agonist as well as 5-HT3, 5-HT7, and 5-HT1D receptor antagonist, and serotonin (5-HT) transporter (SERT) inhibitor [2]. VOR was approved for the treatment of MDD in 2013 by the US Food and Drug Administration (FDA) and the European Medicines Agency (EMA) [3,4]. The drug is available in the form of tablets (Brintellix or Trintellix) in doses of 5 mg, 10 mg, 15 mg, and 20 mg [5]. VOR is also a potential drug candidate for fibromyalgia. Recent research indicates that VOR can attenuate fibromyalgia-like symptoms in mice [6].

To date, only a few liquid chromatography (LC) methods were reported in the literature for the analysis of VOR or its impurities in bulk and pharmaceutical formulations [7,8,9,10,11,12,13,14], human body fluids (serum, plasma, saliva) [15,16,17], rat plasma [18,19,20] and rabbit plasma [21]. LC was commonly applied with diode array (DAD) [7,12,13,14,15,17] and mass spectrometry detection (MS, MS/MS) [7,8,9,10,11,14,15,16,17,18,19,20]. VOR was also analyzed using the voltammetric [22] or electrochemical method [23,24]. The degradation and stability study of VOR has not been studied exactly so far. Diego et al. [7] developed the LC method with the DAD detection to quantify VOR in bulk and tablets. The authors of the above-mentioned work also reported the major oxidative degradation product of VOR as the benzylic alcohol of VOR. There is only one systematic study on the stress degradation behavior of VOR with the characterization of forced degradation products of VOR by LC/MS/MS and NMR [11]. Liu et al. [8] developed the stability-indicating HPLC method to separate and identify potential process-related impurities in VOR. The HPLC method and the fluorescence detection and hydrophilic interaction chromatography (HILIC)-MS method were applied to determine genotoxic impurity from the synthesis route [9]. Dong et al. [10] reported a precolumn derivatization method to determine piperazine trace residues in vortioxetine hydrobromide by HPLC-MS using a C8 Column.

The analysis of VOR was performed mainly using the octadecyl stationary phase. However, according to our earlier study, other chromatographic columns can be successfully applied. For example, the good shape peak and high system efficiency were obtained using the HPLC-DAD method with the Polar RP column and mobile phase containing the addition of DEA [15].

To date, the stability and forced degradation studies of VOR have not been performed sufficiently. The degradation profiles of VOR were presented only for bulk substances. There is no information about potential differences compared to the degradation of the drug in a pharmaceutical formulation. Moreover, broader research on drug quantification and degradation rates under various stress conditions is needed. There here has also been no degradation kinetics study up to now. The above information will be valuable for understanding the chemical stability of VOR and developing a suitable formulation and screening for appropriate storage conditions. To the best of our knowledge, no research presents a fully validated stability-indicating LC-DAD method to determine VOR in the presence of all known degradation products. Diego et al. [7], along with Tiris et al. [12], developed the HPLC-DAD method, which was tested for the VOR analysis in the presence of only its oxidative DPs. No DPs were detected under other stress conditions applied, including the photodegradation study. However, in another research work, the formation of such compounds was proved [11].

The degradation of drugs may result in the loss of drug effectiveness; it can also lead to additional adverse effects due to the formation of toxic DPs. For this reason, it is necessary to the identification of DPs formed during the drug degradation process. Determining the toxicity of detected compounds is necessary to identify their harmful effects on humans, other organisms, and the environment. Many in silico methods were developed to predict the toxicity of chemical compounds. In silico modeling methods are becoming increasingly popular and significant research tools [25]. It is a good alternative to conduct the initial toxicity assessment instead of animal models that have been used for a long time for toxicity testing. In vivo animal tests are time-consuming and constrained by ethical considerations and financial costs. In silico toxicology, on the other hand, is a cheap and fast tool to detect hazards [26]. 

Keeping the above factors in mind, the first purpose of the study was to develop a validated, simple, fast, accurate, and precise stability-indicating HPLC-DAD method for the determination of VOR in the presence of its DPs formed under different stress testing. The method was successfully applied to quantify VOR in bulk and commercial tablets. To the best of our knowledge, we described the fully validated method for the determination of vortioxetine in bulk and tablets in the presence of all known degradation products formed under different stress testing for the first time. Stability studies of vortioxetine under various conditions, including stress tests, have been performed to a greater extent than the previously published research results known from the literature. Moreover, the oxidative and photodegradation kinetic was performed and studied in detail. The structures of the VOR and its degradation products were elucidated by using rapid ultra-high-performance liquid chromatography coupled to the electrospray interface with the quadrupole time-of-flight tandem mass spectrometer (UHPLC/ESI-Q-TOF-MS/MS) method. Finally, an in silico toxicity prediction study of the DPs was performed for the first time. Hence, the presented data in this paper is based on complex and systematic studies on the degradation behavior of VOR. 

## 2. Results and Discussion

### 2.1. HPLC Method Development

**Figure 1:**
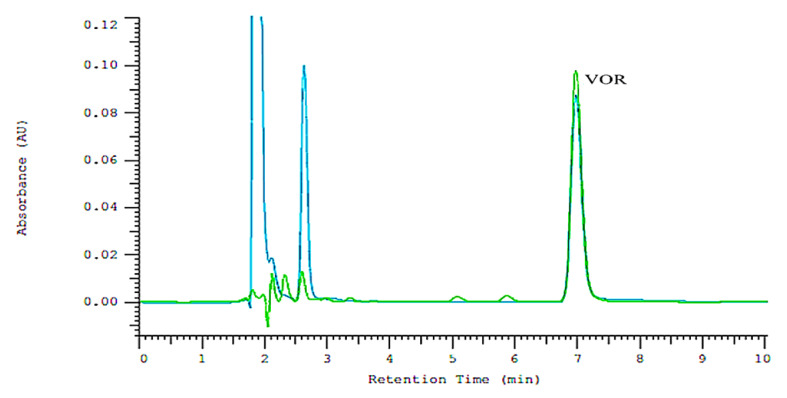
Example of chromatograms obtained for samples after the oxidative (blue color) and photolytic degradation (green color).

In the first step of the study, the appropriate chromatographic system was selected for the HPLC-DAD analysis. The preliminary optimization of chromatographic conditions was necessary to achieve a good separation of VOR and its DPs within a reasonable run time. Synergi Polar RP column, 80 Å, 150 mm × 4.6 mm, 4 μm particle was applied as a stationary phase. As our previous research has shown, the system efficiency obtained on this column is significantly higher than that obtained on the commonly used RP 18 column [27]. In the present study, eluent containing methanol, acetonitrile, acetate buffer pH 3.5, and addiction of diethylamine (DEA) was used as a mobile phase. Applying the stationary phase with π type ligands such as ether-linked phenyl phase—Polar-RP and mobile phase containing the addition of silanol blocker (e.g., DEA) leads to obtaining double protection against interactions between basic aromatic compounds and free silanol groups. The application of such a chromatographic system allows obtaining more symmetrical peaks and high system efficiency, which leads to better separation selectivity. As demonstrated in our previous works concerning the analysis of psychotropic drugs [28], including vortioxetine [15], in systems containing MeOH as an organic modifier, better peak shapes were obtained compared with systems with ACN, but the higher systems efficiency was obtained in eluent systems with ACN. Due to this fact, the mixture of MeOH and ACN in aqueous eluents can be applied, which allows obtaining intermediate A_S_ and N/m values [28]. In the present study, different proportions of ACN and MeOH were tested to provide adequate retention, system efficiency, peak shapes, and selectivity in a short separation time. For further analysis, eluents consisting of MeOH 30% *v*/*v*, ACN 30% *v*/*v*, acetate buffer (pH 3.5) 20% *v*/*v*, double distilled water 20% *v*/*v*, and 0.025 M L^−1^ DEA were selected. The HPLC analyses were performed in the isocratic mode. The optimum wavelength for the determination of VOR was found to be 226 nm and a flow rate of 1 mL/min. The application of the above system allowed obtaining the high system efficiency (N/m = 57162), symmetrical peak (As = 1.19), and good separation selectivity in all the tested cases. The VOR peak was separated from DPs peaks in all forced degradation studies. Figure 1 shows examples of chromatograms obtained for samples after the oxidative (15% H_2_O_2_, duration 6 h) and photolytic degradation (UV 254 nm, duration 16 h). The retention time for VOR was 7.00 min. DPs were eluted before VOR. The total time of a single analysis did not exceed 10 min. The developed HPLC-DAD method was applied to the quantitative analysis of VOR in order to determine the degradation kinetics of VOR. The quantitation of VOR was performed by the external standard calibration.

The developed HPLC-DAD method was validated, considering the requirements of the ICH guidelines [29]. The obtained method is fully stability-indicating due to allowing for the determination of VOR in the presence of all known degradation products resulting from various stressful conditions. Diego et al. [7] and Tiris et al. [12] also developed the HPLC-DAD method to determine VOR. However, the VOR analysis was performed only in the presence of its oxidative DPs. In addition, both methods required the use of an internal standard for quantitative determinations. In the first of the above studies, the retention time (t_R_) of VOR in the developed method was 12 min. Only one product of the oxidative degradation was detected. On the other hand, in the case of the second work, a poor selectivity of separation between the internal standard and the analyte was obtained in the case of the analysis of the sample subjected to the oxidative degradation (it results from the chromatogram presented by the authors).

### 2.2. Method Validation

#### 2.2.1. Linearity

The calibration plot was linear over the investigated concentration range from 1 to 100 µg/mL. The equation of the calibration curve was y = 49,940x + 31,468; the average correlation coefficient r was 0.9999 (Table 1).

#### 2.2.2. Lower Limit of Detection (LLOD) and Lower Limit of Quantification (LLOQ)

The LLOD and LLOQ were found to be 1.32 µg/mL and 3.99 µg/mL, respectively (Table 1).

#### 2.2.3. Accuracy and Precision

To assess the method’s accuracy, the recoveries of VOR were determined. Intraday recoveries were obtained in the range 101.11–101.39% and the inter-day recovery was 100.92 to 101.36%. The precision of the method was calculated as % RSD. The values of the intra-day precision were obtained in the range 0.72–0.91% RSD. The inter-day accuracy was between 0.87 and 1.4% RSD. The intra- and inter-day accuracy and precision results are summarized in Table 2.

#### 2.2.4. Selectivity

Findings from the stress studies indicated that the method could separate VOR from DPs peaks, as shown in Figure 1. Besides, no interference from formulation excipients was found. The peak purity assessment confirmed the selectivity of the method. The peak purity index for vortioxetine was found to be greater than 0.999 in all the cases. 

#### 2.2.5. System Suitability

The system suitability was established by injecting a standard solution (20 µg/mL) and results are as follows: t_R_ = 7.0, A_s_ = 1.19, N/m = 57162, capacity factor = 3.16.

#### 2.2.6. Robustness

The obtained findings are presented in Table 3, indicating that the results remained unaffected by small variations of investigated parameters such as the mobile phase’s column temperature, flow rate, and composition. Thus, these parameters did not significantly affect the determination of the VOR, which indicated that the developed method was robust.

### 2.3. Solution Stability

The stability of VOR in the mobile phase was investigated by analyzing the standard of VOR (20 µg/mL) at 0, 3, 6, 9, 12, and 24 h. Additionally, stability of the stock solution of VOR (1 mg/mL) was also performed. No significant variation in the recovery was observed (Table 4 and Table 5), and also no additional peaks were found in the chromatogram, indicating that VOR was stable in the mobile phase.

### 2.4. Application for Pharmaceutical Formulation

**Figure 2:**
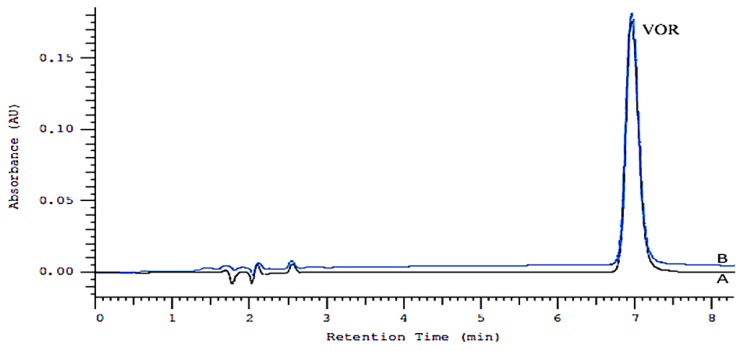
Chromatogram obtained for a standard solution (A—black color) and Brintelix tablets (B—blue color).

The proposed HPLC-DAD method was successfully applied to determine VOR in its dosage form Brintelix tablets 10 mg. Six replicate determinations were performed. Figure 2 illustrates two chromatograms obtained following the assay of a standard solution (A) and Brintelix tablets (B). The found peak purity index was 1.00. The result of the assays undertaken yielded 101.92% (%RSD = 1.11%) of label claim for VORT. The observed concentrations of VOR were found to be 20.38 ± 0.23 µg/mL (mean ± SD). 

### 2.5. Degradation Study

#### 2.5.1. Degradation Tests and Kinetic Study

The study indicated that DPs were formed under photolytic and oxidative stress conditions, whereas VOR was stable under thermal and hydrolytic (i.e., acidic, basic, and neutral) stress conditions. These findings confirm the results obtained by Nagarjuna Chary Ragi et al. [11]. The obtained data were used to calculate VOR concentration at proper time intervals during its forced degradation (Table 6). Forced degradation studies were performed independently for the bulk substance and tablets of VOR. The highest degradation of VOR was obtained in photolysis by UV 254 nm and oxidative stress conditions. For example, the use of 15% H_2_O_2_ causes 46.64–48.76% degradation after 6 h at room temperature. No significant differences were observed between the % degradation of bulk substance and pharmaceutical formulation in all the stress conditions. Therefore, it can be concluded that the formulation ingredients do not significantly affect the degradation rate of VOR.

**Figure 3:**
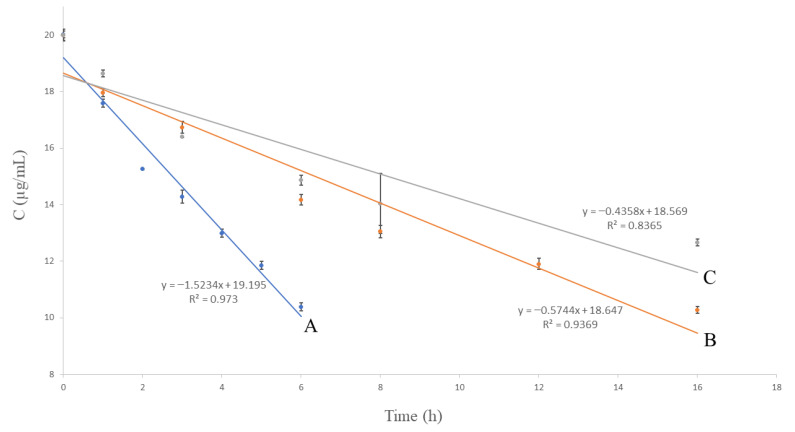
Zero-order kinetic models of the degradation of VOR studied by HPLC; (A) oxidative degradation: 15% H_2_O_2_, RT, (B) photodegradation: UV 254 nm, RT, solution, and (C) photodegradation: UV 254 nm, RT, solid. RT—room temperature (25 ± 2 °C).

**Figure 4:**
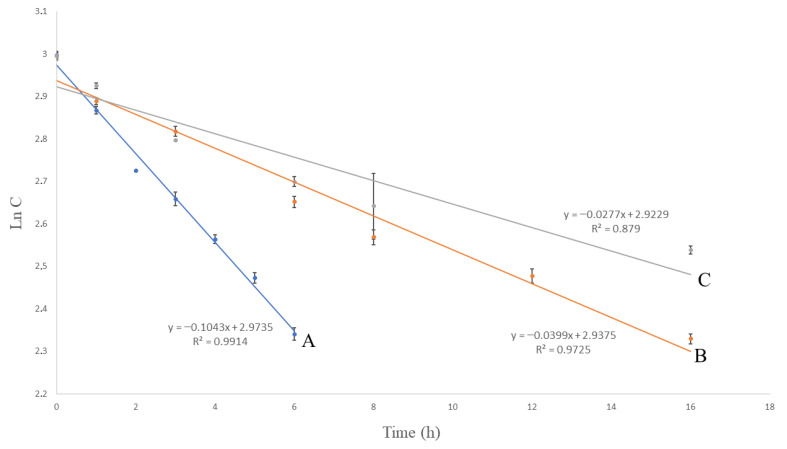
First-order kinetic models of the degradation of VOR studied by HPLC; (A) oxidative degradation: 15% H_2_O_2_, RT, (B) photodegradation: UV 254 nm, RT, solution, and (C) photodegradation: UV 254 nm, RT, solid. RT—room temperature (25 ± 2 °C).

**Figure 5:**
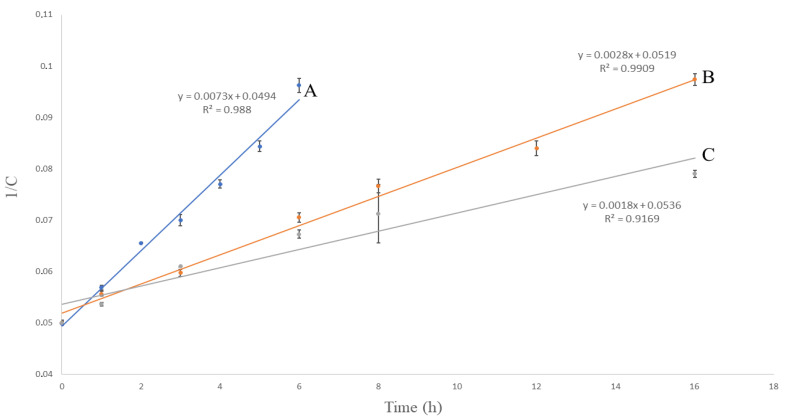
Second-order kinetic models of the degradation of VOR studied by HPLC; (A) oxidative degradation: 15% H_2_O_2_, RT, (B) photodegradation: UV 254 nm, RT, solution, and (C) photodegradation: UV 254 nm, RT, solid. RT—room temperature (25 ± 2 °C).

Various kinetic models were used and compared to examine the rate of the photodegradation and oxidative degradation process. The results are shown in Figure 3, Figure 4 and Figure 5. The correlation coefficient (R^2^) for the first-order kinetics model in the oxidative degradation kinetic study was obtained 0.9914. It was higher than the correlation coefficients derived from zero-order (R^2^ = 0.973) and second-order model fits (R^2^ = 0.988). This suggests that the oxidative degradation process followed the first-order kinetic. In the meantime, the correlation coefficients for the second-order kinetics model in the photodegradation kinetic study were 0.9909 and 0.9169 for the solution and solid form, respectively. The above values were higher than the correlation coefficients derived from zero-order and first-order model fits. This suggests that the photodegradation process followed the second-order kinetic. Having defined the rate constant (*k*), the half-life (*t*_1/2_) and shelf life (*t*_90_) of VOR under each condition were estimated. The kinetic parameters are presented in Table 7.

#### 2.5.2. Identification of DPs by LC-ESI-QTOF-MS

Different types of reactions can induce the chemical degradation of VOR, which causes the gain or loss of its biological activity and the formation of characteristic degradation products. Hence, the drug (VOR) was initially subjected to MS and MS/MS analysis to determine its mass spectral behavior, which can be used for the structural characterization of the DPs.

Based on the obtained results, the most common reaction in the case of VOR is oxidation. It occurs mainly due to present amines and a thioether group, which are prone to oxidation. One of the identified oxidative degradation products could be related to hydroxylation of a methyl group substituent of the aromatic ring. On the other hand, VOR could also undergo comprehensive processes with the formation of its carboxylic acid and a hydroxylation degradation product.

**Figure 6:**
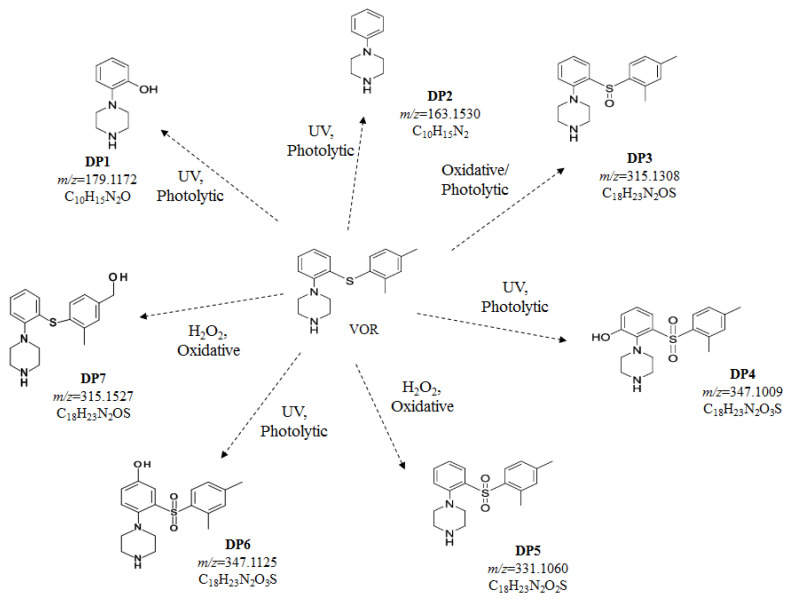
Forced degradation pathway of VOR in tested conditions.

The LC-ESI-Q-TOF-MS/MS method was applied for the identification and structural characterization of vortioxetine and its DPs (Figure S1). The DPs were labeled as DP1-DP7, while LC/HRMS data are summarized in Table 8. The identified DPs were further characterized based on the MS/MS fragmentation patterns. The proposed structures of the observed degradation products are shown in Figure 6. They showed intense [M + H]^+^ ions in the positive ion ESI ionization mode of the analysis. The spectra did not show any adduct ions due to favorable protonation on the nitrogen atom in the piperazine ring, respectively. The proposed fragmentations of VOR and its degradation products DP1-DP7 are summarized in Figure S2. A free radical mechanism can explain the formation of all the DPs under photolytic stress conditions (DP1, DP2, DP4 and DP6). For VOR the protonated mass [M + H]^+^ was observed at *m*/*z* = 299.1961 (C_18_H_23_N_2_S^+^). According to the MS/MS fragmentation pattern (collision energy, 25 eV), the characteristic fragment ions of VOR are *m*/*z* 109.0364 (C_6_H_5_S), 120.1021(C_8_H_10_N), 150.0665 (C_8_H_8_NS), 256.1512 (C_16_H_18_NS).

The [M + H]^+^ ion of DP1 displayed at *m*/z = 179.1172, consistent with the elemental composition of C_10_H_15_N_2_O. The ESI-MS/MS spectrum of the [M + H]^+^ ion (*m*/z = 179.1172) displayed the relevant ions at *m*/z = 136.0763 (C_8_H_10_NO^+^) and *m*/z = 44.0503 (C_2_H_6_N^+^), which are characteristic for the presence of a piperazine ring respectively. This probably suggested bounding to either the phenyl ring or the tertiary amine of the piperazine. The ion at *m*/z = 136.0763 resulted in a peak at *m*/z = 108.0441 (C_7_H_6_NO^+^), by the loss of 28 Da (-C_2_H_4_), which could be explained by the presence of a hydroxy group on the phenyl ring. The formation of a peak at *m*/z = 85.0747 could be illustrated by the loss of C_6_H_6_O from the [M + H]^+^ ion, supporting the presence of an oxygen atom on the phenyl group. The mass value of DP1 was 16 Da higher than that of DP2, hinting that the DP1 may be an oxidized product of DP2. The ion *m*/*z* = 44.0503 is consistent with the piperazine ring in DP1. According to the MS/MS fragmentation pattern (collision energy, 20 eV), the characteristic fragment ions of DP1 are *m*/*z* 44.0503 (C_2_H_6_N), 70.0659 (C_4_H_8_N), 85.0747 (C_4_H_9_N_2_), 92.0488 (C_6_H_6_N), 106.0645 (C_7_H_8_N), 108.0441 (C_7_H_6_NO), 118.0660 (C_8_H_8_N), 136.0763 (C_8_H_10_NO).

The mass spectrum of DP2 is characterized by the [M + H]^+^ ion at *m*/z = 163.1530, and its elemental composition corresponded to C_10_H_15_N_2_. The MS/MS spectrum of the peak at *m*/z = 163.1530 showed an abundant product ion at *m*/z = 120.1052 (C_8_H_10_N^+^), corresponding to the loss of -C_2_H_5_N characteristic for the piperazine ring. The product ion *m*/z = 106.0909 also provides a clue on the attachment of the phenyl group to a piperazine ring. The spectrum also included a peak at *m*/z = 77.0616 (C_6_H_5_^+^), confirming the presence of a phenyl group. Moreover, the fragment ion corresponding to C_2_H_6_N^+^ (*m*/z = 44.0659) was observed in the mass spectrum. The ion *m*/*z* = 44.0659 is consistent with the piperazine ring in DP2. According to the MS/MS fragmentation pattern (collision energy, 15 eV), the characteristic fragment ions of DP2 are *m*/*z* 44.0659 (C_2_H_6_N), 77.0616 (C_6_H_5_), 106.0909 (C_7_H_8_N), 118.0851 (C_8_H_8_N), 120.1052 (C_8_H_10_N), 146.0786 (C_10_H_12_N). The homolytic cleavage of the C–S bond of the phenyl piperazine moiety in VOR led to the formation of DP1 (*m*/z = 179.1172) and DP2 (*m*/z = 163.1530), where the former is formed by hydrogen radical attachment and the latter by hydroxyl radical attachment to the phenyl group.

The mass spectrum of DP3 (under oxidative/photolytic conditions) is characterized by the [M + H]^+^ ion at *m*/*z* = 315.1308, corresponding to the formula C_18_H_23_N_2_OS. Moreover, we observed the DP7 (under oxidative conditions) at *m*/*z* = 315.1527 (C_18_H_23_N_2_OS+). The molecular weight of DP3 along with DP7 is 16 Da higher than that of VOR, suggesting the presence of an oxygen atom. The acquired MS/MS spectra proved that the precursor ion characterized for DP3 at *m*/*z* = 315.1308 was fragmented to *m*/*z* = 256.1594 (-C_2_H_5_NO) and *m*/*z* = 242.1397 (-C_3_H_7_NO) respectively. These fragment ions are identical to the characteristic ions of the VOR fragmentation pattern. Moreover, the formation of these two ions may be explained by the opening of the piperazine ring. Additionally, the [MH-C_8_H_10_]^+^, *m*/*z* = 209.1160 is consistent with the presence of a dimethyl phenyl group on the sulfur atom. Loss of H_2_O resulted in a more stable ion at *m*/*z* = 191.0128.

In the literature, regarding the DP at *m*/*z* = 315, there are various proposals of the identified structure, mainly due to the insufficient structure indicative of the fragment ions to confirm the oxidation site. Ragi et al. [11] identified and characterized the forced degradation products of VOR by LC/MS/MS and NMR. They synthesized the product to confirm the structure of mono-oxidized DP of VOR. With the use of HR-ESIMS, they observed the [M + H]^+^ ion at *m*/*z* = 315.1519. On the other hand, Deigo et al. [7] identified the oxidative DPs of VOR in bulk. Based on the MS/MS data, they proposed a different structure for this oxidized product (*m*/*z* = 315.3). The MS/MS spectrum of the mono-oxidized product reported by them was found to be similar to the MS/MS spectrum of DP7 obtained in the current study. This DP was also reported as the metabolites (Lu AA25790 and Lu AA39835) of VOR with *m*/*z* of the molecular ion of 315 [17]. 

According to our results and the MS/MS fragmentation pattern (collision energy, 25 eV), the characteristic fragment ions of DP3 are *m*/*z* 44.0673 (C_2_H_6_N), 56.0697 (C_3_H_6_N), 72.0678 (C_4_H_10_N), 94.0910 (C_6_H_8_N), 106.0929 (C_7_H_8_N), 120.1103 (C_8_H_10_N), 136.0531 (C_7_H_6_NS), 138.0118 (C_7_H_8_NS), 148.0555 (C_8_H_6_NS), 162.0729 (C_9_H_8_NS), 191.0128 (C_10_H_11_N_2_S), 209.1160 (C_10_H_13_N_2_OS), 242.1397 (C_15_H_16_NS), 256.1594 (C_16_H_18_NS). The ions *m*/*z* = 44.0673, *m*/*z* = 56.0697 and *m*/*z* = 72.0678 are consistent with the piperazine ring in DP3. On the other hand, for DP7 and the characteristic fragmentation pattern (collision energy, 30 eV), the fragment ions are 74.0845 (C_4_H_12_N), 106.1101 (C_7_H_8_N), 120.1047 (C_8_H_10_N), 136.0417 (C_7_H_6_NS), 191.0902 (C_10_H_11_N_2_S), 256.2938 (C_16_H_18_NS), respectively.

The mass spectra of DP4 and DP6 the [M + H]^+^ ion at *m*/*z* = 347.1119 and *m*/*z* = 347.1125, respectively, correspond to the formula C_18_H_23_N_2_O_3_S. The LC-ESI-MS/MS spectra of the [M + H]^+^ ions for both DP4 and DP6 showed the [MH-C_2_H_5_N]^+^ ion (*m*/*z* = 304.0676 and *m*/*z* = 304.1010) and other common fragment ions that were observed for DP4 at *m*/*z* 44.0660, 56.0682, 70.0860, indicating the presence of a piperazine ring. The fragmentation pattern of DP6 was found to be similar to that of DP4, suggesting the −OH group on the phenyl group may be away from the other functional groups. Based on its characteristic fragmentation pattern, the fragment ions of DP4 (collision energy, 20 eV) are 44.0660 (C_2_H_6_N), 56.0682 (C_3_H_6_N), 70.0860 (C_4_H_8_N), 85.0375 (C_4_H_9_N_2_), 105.0971 (C_7_H_7_N), 136.0504 (C_7_H_6_NS), 304.0676 (C_16_H_18_NO_3_S). The ions *m*/*z* = 44.0660 and *m*/*z* = 56.0682 are consistent with the piperazine ring in DP4. On the other hand, for DP6 (collision energy, 25 eV), they are 56.0674 (C_3_H_6_N), 70.1192 (C_4_H_8_N), 84.3469 (C_4_H_8_N_2_), 106.0865 (C_7_H_8_N), 120.3363 (C_8_H_10_N), 134.0822 (C_7_H_4_NS), 149.1328 (C_8_H_5_NS), 164.1592 (C_8_H_6_NOS), 205.0757 (C_10_H_9_N_2_OS), 223.0854 (C_10_H_11_N_2_O_2_S), 304.1010 (C_16_H_18_NO_3_S). The ion *m*/*z* = 56.0682 is consistent with the piperazine ring in DP6. The formation of the photolytic degradation product DP4 and DP6 could be explained by the oxidation of the phenyl ring and sulfur atoms.

The mass spectrum for DP5 is characterized to the [M + H]^+^ ion at *m*/z = 331.1060, corresponding to the formula C_18_H_23_N_2_O_2_S. The elemental composition suggested the presence of two additional oxygen atoms in DP5 compared to that of VOR. A likely structure for the formation of DP4 could occur by the oxidation of the sulfur atom forming the corresponding sulfone. The LC-ESI-MS/MS spectrum of the [M + H]^+^ peak of DP5 showed *m*/z = 288.1306, corresponding to the loss of C_2_H_5_N (43 Da) from [M + H]^+^ ion, which is characteristic of the piperazine ring. Based on its characteristic fragmentation pattern (collision energy, 20 eV), the fragment ions of DP5 are 44.0652 (C_2_H_6_N), 77.7323 (C_4_H_15_N), 91.0793 (C_7_H_7_), 106.0926 (C_7_H_8_N), 119.1755 (C_8_H_9_N), 136.0527 (C_7_H_6_NS), 140.0436 (C_6_H_6_NOS), 162.3383 (C_9_H_8_NS), 178.0349 (C_9_H_8_NOS), 190.0881 (C_10_H_10_N_2_S), 288.1306 (C_16_H_18_N_2_O_2_S).

Nuclear magnetic resonance (NMR) spectroscopy and LC-MS/MS could be the powerful tool widely used in VOR DPs searching and identification. However, in our case, we cannot receive such a huge amount of suitable samples of degradation products necessary for the NMR measurements.

### 2.6. In Silico Toxicity Studies

**Figure 7:**
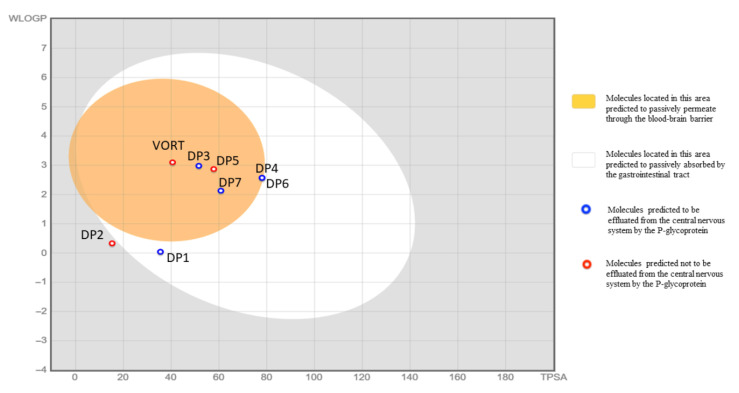
The BOILED-Egg model: evaluation of passive gastrointestinal absorption (HIA) and brain barrier penetration (BBB) in the function of the position of the molecules in the WLOGP (lipophilicity parameter) versus TPSA (total polar surface area).

The experimental methods for predicting the compound’s pharmacokinetics and toxicity are tedious and time-consuming tasks. Thus, the computational approaches could be used as a tool to develop alternative methods for toxicity prediction. SwissADME was applied as a tool to assess the physicochemical properties and pharmacokinetics of VOR DPs. The results are presented in Table 9. Figure 7 shows the BOILED-Eggmodel that allows evaluating the passive gastrointestinal absorption (HIA) and brain barrier penetration (BBB) in the function of the position of the molecules in the WLOGP (lipophilicity parameter) versus TPSA (total polar surface area). All the degradation products are more polar than vortioxetine and are more soluble and less lipophilic. DPs may affect hepatic isozymes (as inhibitors). There is, therefore, potential risk of pharmacokinetic interactions with substances metabolized by these enzymes. The BOILED-Egg model indicates that DP3, DP4, DP5, DP6, DP7 can passively permeate through the blood-brain barrier. The model also predicts a high absorption of DPs from the gastrointestinal tract except for DP2. Lazar toxicity predictions indicate the potential risk of mutagenic and carcinogenic effects of some DPs (from DP2 to DP7; Table S1).

In vivo assessment toxicity of DPs is of great importance and should be presented in the future in a separate study.

## 3. Materials and Methods

### 3.1. Chemicals and Reagents

Acetonitrile (ACN), methanol (MeOH) of chromatographic quality, diethylamine (DEA), formic acid (98–100%), acetic acid (99–100%), ammonium formate, sodium acetate, and water for LC-MS were purchased from Merck (Darmstadt, Germany). Ammonium (25%), ammonium chloride, 30% hydrogen peroxide of trace analysis grade, vortioxetine standard (purity = 99.8%) were obtained from Sigma Aldrich (St Louis, MO, USA). Brintelix (vortioxetine 10 mg) was purchased from Lundbeck. Water for LC-DAD analysis was double distilled.

### 3.2. Preparation of Stock Solution and Working Solutions

The stock standard solution of VOR was prepared in MeOH at a concentration of 1 mg/mL by dissolving an amount of vortioxetine hydrobromide corresponding to 50 mg of the free base in 50 mL of MeOH. The solution was stored at −20 °C in a glass vial, protected from light.

The working standard solutions of VOR were prepared from the stock solutions immediately before the analysis by diluting the above-mentioned stock solution in MeOH before the analysis.

### 3.3. Apparatus and LC Conditions

HPLC-DAD and LC-ESI-QTOF-MS performed the chromatographic analyses of vortioxetine and their degradation products. The LC conditions are described below. 

#### 3.3.1. HPLC-DAD Conditions

The HPLC analyses were performed using the liquid chromatograph LaChrom Elite (Merck) equipped with a column oven L-7350, a solvent degasser L-7612, an autosampler, and a DAD detector. The analyses were conducted at 22 °C with an eluent flow rate of 1.0 mL/min. The DAD detector was set in the 200–400 nm range. The qualitative and quantitative analyses were performed at 226 nm. The injection volume was 20 μL. The Synergi Polar RP column, 80 Å, 150 mm × 4.6 mm, 4 μm particle (Phenomenex, Torrance, CA, USA)), was applied as the stationary phase. The eluent consisted of MeOH 30% *v*/*v*, ACN 30% *v*/*v*, acetate buffer (pH 3.5) 20% *v*/*v*, double distilled water 20% *v*/*v*, and 0.025 M L^−1^ DEA. The HPLC analyses were performed in the isocratic mode. The retention time for vortioxetine was 7.00 min. The chromatographic data were acquired and further processed with the EZchrom Elite software. The peak purity was confirmed by comparing the UV spectra obtained for vortioxetine in tested samples with the standard spectra. The peak purity index for vortioxetine was found to be greater than 0.999 in all the cases.

#### 3.3.2. LC-ESI-Q-TOF-MS Conditions

The determination and identification of VOR and its degradation products were carried out using a UHPLC Agilent 1290 Series system (Agilent Technologies, Waldbronn Germany) equipped with an ESI interface, a 6540 UHD accurate mass Q-TOF detector, and Mass Hunter software for data collection and instrumental control. The mass spectrometer was calibrated before the analysis using the manufacturer’s calibration solution. Chromatographic C18 column (4.6 mm × 100 mm, 1.8 µm, Agilent Technologies, Germany) was maintained at 25 ± 0.5 °C. The injected sample volume was 10 µL, while the mobile phase was composed of ACN and 0.1% HCOOH (70:30) dosed at a flow rate of 0.4 mL/min. The retention time for VOR was 8.61 min. Quadrupole time-of-flight mass spectrometric analyses were performed using the electrospray ion source operating in the positive ion mode (ESI(+)), with the following set of operation parameters: the capillary voltage (CV), 3.5 kV; the octopole voltage (OV), 750 V; the skimmer voltage (SV), 45 V; the drying gas temperature (DGT), 260 °C; the shielding gas temperature (SGT), 305 °C; the fragmentor voltage (FV), 175 V. The Q-TOF and information-dependent acquisition scan operated with a mass range of 40 to 400 *m*/*z*. Nitrogen was used as drying (6 L/min) and nebulizing (35 psig) gas. Nitrogen was used as the collision gas, and the collision energy used was 15–30 eV. High-purity nitrogen gas was used for the nebulizer/DuosprayTM(Agilent Technologies, Waldbronn, Germany) and curtain gases. The data acquisition and processing were carried out using the MassHunter Workstation software (B.04.01, Agilent Technologies, Waldbronn, Germany).

#### 3.3.3. Method Validation

The proposed HPLC-DAD method was validated with respect to the International Conference on Harmonization (ICH) Q2 (R1) guideline (ICH, 2005). Validation parameters included: linearity, the lower limit of detection (LLOD) and the lower limit of quantification (LLOQ), selectivity, accuracy, precision, robustness, and system suitability.

#### 3.3.4. Linearity

The calibration curve was prepared by analyzing standard solutions in triplicate at seven concentrations, ranging from 1 to 100 μg/mL. The calibration curves were obtained by plotting the peak area versus the concentration. 

#### 3.3.5. Lower Limit of Detection (LLOD) and Lower Limit of Quantification (LLOQ)

LLOD and LLOQ were calculated according to the formulas: LLOD = 3.3 (SD/S) and LLOQ = 10 (SD/S), where S is the slope of the calibration curve and SD is the standard deviation of response (Table 1).

#### 3.3.6. Accuracy and Precision

The method’s accuracy was tested by performing recovery studies at three different concentration levels, 80%, 100%, and 120%, by spike known quantities of the drug analyte, and recovery percentages were calculated. The method’s precision was calculated as % relative standard deviation (% RSD). Both precision and accuracy were assessed by calculating the intra-day and inter-day variation. In the intra-day studies, drug solutions were analyzed on the same day (n = 6). In the inter-day studies, samples were analyzed on three consecutive days (n = 18).

#### 3.3.7. Selectivity

The method’s selectivity was assessed by subjecting VOR to various stress conditions (oxidative, photolytic, hydrolytic, thermal degradation) to demonstrate the separation between VOR and its DPs. A possible interference due to excipients present in the commercial tablets was also evaluated. The DAD detector was applied to assess the peak purity to confirm that there were no co-eluting compounds.

#### 3.3.8. System Suitability

The system suitability was established by injecting a standard solution (20 µg/mL of VOR); next, chromatographic parameters such as retention, the capacity factor, the system efficiency, and the peak symmetry were assessed. The system efficiency was expressed as theoretical plates number per meter (N/m) according to US Pharmacopeia. The peak symmetry was expressed as an asymmetry factor (As).

#### 3.3.9. Robustness

The robustness of an analytical method measures its capacity to remain unaffected by small but deliberate variations in the method parameters. Different variations in the mobile phase composition (concentration of MeOH, ACN, water, and acetate buffer), the column temperature (±2 °C), and the flow rate (±0.1 units) were examined. Recoveries and % RSD were calculated as comparison parameters. The results are presented in Table 3.

### 3.4. Solution Stability

The stability of VOR in the mobile phase (20 µg/mL) and the stock solution (1 mg/mL) of VOR (1 mg/mL) were investigated. The analysis of VOR in the mobile phase exposure at room temperature (25 ± 2 °C) was performed after 0, 3, 6, 9, 12, and 24 h (Table 4). The stability of the stock solution of VOR (1 mg/mL) exposure at room temperature (25 ± 2 °C), 4 °C, and −20 °C after 24 h, two weeks and 1 year, respectively, was also conducted (Table 5).

### 3.5. Forced Degradation Study

Stress degradation studies of VOR were carried out on the bulk drug and tablets, considering the ICH guidelines Q1A (R2) [30].

Forced degradation studies were performed independently for the bulk substance and pharmaceutical formulation (tablets) of VOR. As for the stock solutions, the two above-mentioned forms of VOR were prepared in MeOH at the concentration 1 mg/mL. In the case of tablets, the equivalent of 10 mg of VORT from Brintelix formulation was transferred to 10 mL volumetric flask; after the addition of MeOH, it was extracted by a shaker. The obtained suspension was centrifuged and next used as a stock solution. The working solutions were prepared by diluting stock solutions using the proper solvent to obtain a final concentration. The obtained solutions were subjected to various stress conditions. The samples obtained under each forced degradation condition were diluted appropriately with mobile phase to get a final concentration of 20 μg/mL.

The % degradation was calculated according to the following formula:

(1) $$\%\;\mathrm{degradation}=\frac{\mathrm{area}\;\mathrm{of}\;\mathrm{unstressed}-\mathrm{area}\;\mathrm{of}\;\mathrm{stressed}}{\mathrm{area}\;\mathrm{of}\;\mathrm{unstressed}}\;\times \;100\%$$

#### 3.5.1. Photodegradation

##### Normal Light

The process involved 1 mL of the stock solution being exposed to normal white light for 48 h at room temperature (25 ± 2 °C). Then volume was made up with the mobile phase to achieve a final concentration of 20 μg/mL

##### UV Light 245 nm

The process involved 1 mL of stock solution being exposed to UV 245 nm at room temperature (25 ± 2 °C). Then, the volume was made up with the mobile phase to achieve a final concentration of 20 μg/mL.

After, 1 mg of VOR in the solid-state was spread in a Petri plate and was exposed to UV 245 nm at room temperature (25 ± 2 °C). Then, the substance was dissolved in the mobile phase to achieve a final concentration of 20 μg/mL.

##### UV Light 366 nm

The process involved 1 mL of stock solution being exposed to UV 366 nm at room temperature (25 ± 2 °C) for 24 h. Then, the volume was made up with the mobile phase to achieve a final concentration of 20 μg/mL.

After, 1 mg of VOR in the solid-state was spread in a Petri plate and was exposed to UV 366 at room temperature (25 ± 2 °C) for 24 h. Then, the substance was dissolved in the mobile phase to achieve a final concentration of 20 μg/mL.

#### 3.5.2. Thermal Degradation

In order to carry out the thermal degradation of bulk substance, 1 mg of VOR was spread in a Petri plate and heated at 100 °C for 48 h. Next, the substance was allowed to attend the ambient temperature. Then, the substance was dissolved with the mobile phase to achieve a final concentration of 20 μg/mL. For the thermal degradation of VOR in pharmaceutical formulations, 20 tablets (Brintelix, 10 mg) were heated at 100 °C for 48 h. Next, the tablets were allowed to attend the ambient temperature and prepared as described below (look section “Tablet Assay Preparation”).

#### 3.5.3. Acid Degradation

Acid degradation was performed at 70 °C by adding 1 mL of 2 M HCl to 1 mL of the stock solution of VOR, next neutralized by 2 M NaOH after 72 h. Then, the solution was diluted with the mobile phase to achieve a final concentration of 20 μg/mL.

#### 3.5.4. Alkali Degradation

Alkali degradation was performed at 70 °C by adding 1 mL of 2 M NaOH to 1 mL of stock solution of VOR, next neutralized by 2 M HCl after 72 h. Then, the solution was diluted with the mobile phase to achieve a final concentration of 20 μg/mL.

#### 3.5.5. Neutral Degradation

Neutral degradation was performed by adding 1 mL of water to 1 mL of the stock solution of VOR and the exposure at 70 °C for 72 h. Then, the solution was diluted with the mobile phase to achieve a final concentration of 20 μg/mL.

#### 3.5.6. Oxidative Degradation

Oxidative degradation was performed at room temperature by adding 1 mL of 15% H_2_O_2_ to 1 mL of the stock solution of VOR. After the appropriate time, the solution was diluted with the mobile phase to achieve a final concentration of 20 μg/mL and directly injected into the HPLC system.

Samples for neutral, acidic, alkali, oxidative, and thermal degradation were kept in the dark to avoid light’s possible effect.

### 3.6. Degradation Kinetics Study

The quantitative analysis of VOR in the tested samples was performed using DAD detection at wavelength 226 nm. The obtained calibration curve was used to determine the degradation kinetics of VOR in the tested conditions (Table 7). VOR is susceptible to oxidative and photolytic degradation. For this reason, the kinetic studies under this condition were carried out. The kinetic model that best describes the reaction was obtained by the substitution method. 

The first-order and second-order kinetic equations were applied for the calculation of the degradation kinetics parameters: the rate constant (k), the half-life (t_1/2_), and time left for 90% potency (t_90_) according to the undermentioned formulas.

For first-order degradation kinetic:

lnC = ln C_0_ − kt (2)

(3) $$t_{1/2}=\frac{0.693}{k}$$

(4) $$t_{90}=\frac{0.105}{k}$$

For second-order degradation kinetic:

(5) $$\frac{1}{C}=\frac{1}{C_{0}}+\mathrm{kt}$$

(6) $$t_{1/2}=\frac{1}{{\mathrm{kC}}_{0}}$$

(7) $$t_{90}=\frac{1}{9{\mathrm{kC}}_{0}}$$

where:

C_0_—the concentration in time 0,

C—is the remaining concentration.

### 3.7. Tablet Assay Preparation

We accurately weighed and crushed 20 tablets (Brintelix 10 mg) into homogenous powder. A quantity of powder equivalent to one tablet containing 10 mg of vortioxetine was transferred into a 50 mL volumetric flask. After filling up to volume with MeOH, the resulting solution was mechanically shaken for 15 min. Next, aliquots of the solution were transferred into a 10 mL volumetric flask diluted to the appropriate volume with a mobile phase. Before analysis, it was shaken for 10 min and filtered through 0.22 μm PVDF syringe filters.

### 3.8. In Silico Toxicity Studies

The physicochemical and pharmacokinetic properties ADME (Absorption, Distribution, Metabolism, Excretion), as well as the toxicity of the DPs, were predicted along with a massive database on the swiss ADME/T web server (http://www.swissadme.ch/, accessed on 28 November 2021), which can hypothesize compounds properties with high-precision [31]. A predictive toxicology framework called lazar was also applied [32] using a web server (https://lazar.in-silico.ch/predict, accessed on 28 November 2021). A BOILED-Egg (Brain or IntestinaL EstimateD permeation method) [33] was applied as a model to predict the gastrointestinal absorption and brain penetration of small molecules. This model works by computing the lipophilicity and polarity of small molecules.

## 4. Conclusions

The developed HPLC-DAD method is simple, fast, accurate, precise, and stability-indicating. The validation of the method proved that the method is suitable for the determination of VOR in bulk and tablet formulation without any interference from the potential degradation product of VOR. The method can be successfully applied for routine analyses and quality control laboratories for stability studies of VOR tablets or assay of VOR tablets from stability batches.

The oxidative and photolytic degradation kinetics studies were carried out. Under tested conditions, the reactions followed first-order and second-order kinetics for oxidative degradation and photodegradation, respectively. The half-life and shelf life of VOR under each condition were determined based on rate constants. These parameters are important for the process control and formulation development along with compliance with the appropriate storage conditions for bulk substance and pharmaceutical preparations. Potential degradation products were detected by the LC-ESI-QTOF-MS method.

A total of seven degradation products were identified in different conditions. The drug (VOR) and its DPs were separated by UHPLC and characterized by the ESI-Q-TOF-MS/MS analysis. The proposed fragmentation pathway of the drug and its DPs could be used in future evaluations to characterize process-related impurities and metabolites of VOR.

In silico ADME and toxicity studies indicated that DPs may have high GI absorption in most cases, can penetrate the blood-brain barrier, and may influence the activity of some liver enzymes. Some of them also have potential mutagenic and cancerogenic activity; however, the confirmation or rejection of these predictions requires appropriate experimental studies.

## Figures and Tables

**Table 1:** Parameters of calibration curves for quantitative analysis of VOR: calibration curves’ equations, concentration range, regression coefficient (r), the lower limit of detection (LLOD), the lower limit of quantitation (LLOQ).

Parameter	Value
Linearity range (µg/mL)	1–100
Regression equation	y = 49,940x + 31,468
Slope	49,940
Intercept	31,468
r	0.9999
LLOD (µg/mL)	1.32
LLOQ (µg/mL)	3.99

**Table 2:** Validation data.

% of Target Levels	Concentration Added (µg/mL)	Intra-Day (n = 6)		Inter-Day (n = 18)	
		Recovery (%)	%RSD	Recovery (%)	%RSD
80	16	101.23	0.72	101.36	0.87
100	20	101.39	0.91	101.26	1.4
120	24	101.11	0.77	100.92	1.28

**Table 3:** Robustness studies of VOR (n = 6).

Conditions	Recovery (%)	%RSD
Column temperature: 20 °C	101.88	0.63
Column temperature: 24 °C	101.52	0.27
Flow rate: 0.9 mL/min	103.02	1.72
Flow rate: 1.1 mL/min	98.97	0.74
Organic phase composition: MeOH 28% *v*/*v*, ACN 32% *v*/*v*	100.56	0.49
Organic phase composition: MeOH 32% *v*/*v*, ACN 28% *v*/*v*	101.17	0.77
Organic phase composition: MeOH 31% *v*/*v*, ACN 31% *v*/*v*	100.22	0.89
Organic phase composition: MeOH 29% *v*/*v*, ACN 29% *v*/*v*	101.81	0.83
The content of the acetate buffer: 18% *v*/*v*	102.35	0.58
The content of the acetate buffer: 22% *v*/*v*	101.28	0.97

**Table 4:** Stability of VOR in the mobile phase (RT, n = 6).

Time (h)	Recovery (%)	SD
0	101.14	0.52
4	101.70	0.62
8	102.33	0.74
12	101.46	0.92
24	100.83	0.95
Mean:	101.49	0.75

RT—room temperature (25 ± 2 °C).

**Table 5:** Stability of VOR in the stock solution (n = 6).

Conditions	Time	Recovery (%)	SD
RT	48 h	101.51	0.99
4 °C	14 days	102.24	1.26
−20 °C	12 months	101.51	0.99

RT—room temperature (25 ± 2 °C).

**Table 6:** Stress degradation data of the VOR.

Tested Form of VOR	Stress Type	Stress Condition	Exposed Conditions	Duration	Calculated VOR Concentration * (µg/mL) ± SD	Recovery * (%) ± SD	% Degradation * ± SD	VOR Peak Purity Index	Remarks
STD	Acid hydrolysis, solution	2 M HCl	70 °C	72 h	19.98 ± 0.12	99.92 ± 0.58	0.87 ± 0.45	1.0000	No degradation was observed
TAB					20.18 ± 0.1	100.90 ± 0.49	0.24 ± 0.38	1.0000	
STD	Base hydrolysis, solution	2 M NaOH	70 °C	72 h	20.0 ± 0.13	100.00 ± 0.65	0.78 ± 0.51	1.0000	
TAB					20. 15 ± 0.1	100.77 ± 0.5	0.37 ± 0.39	1.0000	
STD	Neutral hydrolysis, solution	H_2_O	70 °C	72 h	19.97 ± 0.09	99.84 ± 0.44	0. 95 ± 0.35	1.0000	
TAB					20.03 ± 0.12	100.17 ± 0.61	0.94 ± 0.39	1.0000	
STD	Thermal, solid form	Solid	100 °C	48 h	19.91 ± 0.08	99.56 ± 0.42	0.54 ± 0.33	1.0000	
TAB					19.99 ± 0.1	99.97 ± 0.5	1.13 ± 0.39	1.0000	
STD	Photodegradation, solid	Normal white light	RT	48 h	20.16 ± 0.07	100.78 ± 0.36	0.87 ± 0.28	1.0000	
	Photodegradation, solution				20.07 ± 0.19	100.37 ± 0.96	1.26 ± 0.75	1.0000	
TAB	Photodegradation, solution				20.17 ± 0.05	100.84 ± 0.23	−0.8 ± 0.19	1.0000	
STD	Photodegradation, solid	UV 254 nm	RT	16 h	12.66 ± 0.12	63.28 ± 0.58	36.64 ± 0.45	1.0000	Five degradation products: DP1, DP2, DP3, DP4, DP6
	Photodegradation, solution				10.27 ± 0.12	51.35 ± 0.62	48.02 ± 0.42	1.0000	
TAB	Photodegradation, solution				10.03 ± 0.1	50,17 ± 0.52	48.88 ± 0.35	0.999	
STD	Photodegradation, solid	UV 366 nm	RT	24 h	18.28 ± 0.2	91.39 ± 1	9. 08 ± 0.79	1.0000	
	Photodegradation, solution				18.23 ± 0.19	91.16 ± 0.94	9.29 ± 0.73	1.0000	
TAB	Photodegradation, solution				18.78 ± 0.03	93.88 ± 0.16	6.12 ± 0.12	1.0000	
STD	Oxidation, solution	15% H_2_O_2_	RT	6 h	10.39 ± 0.15	51.94 ± 0.75	46.64 ± 0.59	1.0000	Three degradation products: DP3, DP5, DP7
TAB					10.06 ± 0.11	50.29 ± 0.55	48.76 ± 0.43	1.0000	

STD—bulk substance, TAB—tablets, RT—room temperature (25 ± 2 °C), * n = 3.

**Table 7:** Results of degradation kinetics study (n = 3).

Degradation Conditions	Duration (h)	k (h^−1^) ^a^ ± SD	t_1/2_ (h) ^b^ ± SD	t_90_ (h) ^c^ ± SD
Oxidative degradation: 15% H_2_O_2_, RT	0, 1, 2, 3, 4, 5, 6	0.1043 ± 0.0026	6.64 ± 0.17	1.01 ± 0.03
Photodegradation: UV 254 nm, RT, solution	0, 1, 3, 6, 8, 12, 16	0.0028 ± 0.0001	17.86 ± 0.36	1.98 ± 0.04
Photodegradation: UV 254 nm, RT, solid	0, 1, 3, 6, 8, 16	0.0018 ± 0.0001	27.78 ± 0.94	3.09 ± 0.1

^a^ Rate constant per hour; ^b^ Half-life; ^c^ Time left for 90% potency; RT—room temperature (25 ± 2 °C).

**Table 8:** LC-ESI-QTOF-MS data of VOR and its DPs.

Compound/ Degradation Product	Stress Conditions	Chemical Formula	Molecular Ion [M + H]^+^ *m*/*z*	MS/MS Fragment Ions [M + H]^+^ (Observed Mass *m*/*z*)	Calculated Mass *m*/*z* (Error ppm)
VOR	-	C_18_H_23_N_2_S	299.1961		299.1981 (−6.68)
				109.0364 (C_6_H_5_S)	109.0368 (−3.67)
				120.1021 (C_8_H_10_N)	120.1011 (8.32)
				150.0665 (C_8_H_8_NS)	150.0659 (3.99)
				256.1512 (C_16_H_18_NS)	256.1508 (1.56)
DP1	Photolytic	C_10_H_15_N_2_O	179.1172		179.1181 (−5.02)
				44.0503 (C_2_H_6_N)	44.05017 (2.95)
				70.0659 (C_4_H_8_N)	70.0663 (−5.71)
				85.0747 (C_4_H_9_N_2_)	85.0742 (5.88)
				92.0488 (C_6_H_6_N)	92.0493 (−5.43)
				106.0645 (C_7_H_8_N)	106.0651 (−5.66)
				108.0441 (C_7_H_6_NO)	108.0448 (−6.48)
				118.0660 (C_8_H_8_N)	118.0658 (1.69)
				136.0763 (C_8_H_10_NO)	136.0759 (2.94)
DP2	Photolytic	C_10_H_15_N_2_	163.1530		163.1541 (−6.74)
				44.0659 (C_2_H_6_N)	44.0658 (2.26)
				77.0616 (C_6_H_5_)	77.0608 (10.38)
				106.0909 (C_7_H_8_N)	106.0914 (−4.71)
				118.0851 (C_8_H_8_N)	118.0856 (−4.23)
				120.1052 (C_8_H_10_N)	120.1054 (−1.66)
				146.0786 (C_10_H_12_N)	146.0791 (−3.42)
DP3	Oxidative/Photolytic	C_18_H_23_N_2_OS	315.1308		315.1317 (−2.86)
				44.0673 (C_2_H_6_N)	44.0668 (11.35)
				56.0697 (C_3_H_6_N)	56.0694 (5.35)
				72.0678 (C_4_H_10_N)	72.0683 (−6.94)
				94.0910 (C_6_H_8_N)	94.0915 (−5.31)
				106.0929 (C_7_H_8_N)	106.0923 (5.65)
				120.1103 (C_8_H_10_N)	120.1107 (−3.33)
				136.0531 (C_7_H_6_NS)	136.0535 (−2.94)
				138.0118 (C_7_H_8_NS)	138.0121 (−2.17)
				148.0555 (C_8_H_6_NS)	148.0554 (0.67)
				162.0729 (C_9_H_8_NS)	162.0731(−1.23)
				191.0128 (C_10_H_11_N_2_S)	191.0134 (−3.14)
				209.1160 (C_10_H_13_N_2_OS)	209.1164 (−1.91)
				242.1397 (C_15_H_16_NS)	242.1391 (2.48)
				256.1594 (C_16_H_18_NS)	256.1598 (−1.56)
DP4	Photolytic	C_18_H_23_N_2_O_3_S	347.1119		347.1121 (−0.58)
				44.0660 (C_2_H_6_N)	44.0663 (−6.81)
				56.0682 (C_3_H_6_N)	56.0679 (5.35)
				70.0860 (C_4_H_8_N)	70.0867 (−9.99)
				85.0375 (C_4_H_9_N_2_)	85.0371 (4.70)
				105.0971 (C_7_H_7_N)	105.0978 (−6.66)
				136.0504 (C_7_H_6_NS)	136.0507 (−2.20)
				304.0676 (C_16_H_18_NO_3_S)	304.0681 (−1.64)
DP5	Oxidative	C_18_H_23_N_2_O_2_S	331.1060		331.1064 (−1.21)
				44.0652 (C_2_H_6_N)	44.0647 (11.35)
				77.7323 (C_4_H_15_N)	77.7326 (−3.86)
				91.0793 (C_7_H_7_)	91.0798 (−5.49)
				106.0926 (C_7_H_8_N)	106.0931 (−4.71)
				119.1755 (C_8_H_9_N)	119.1758 (−2.52)
				136.0527 (C_7_H_6_NS)	136.0533 (−4.41)
				140.0436 (C_6_H_6_NOS)	140.0441 (−3.57)
				162.3383 (C_9_H_8_NS)	162.3389 (−3.70)
				178.0349 (C_9_H_8_NOS)	178.0344 (2.81)
				190.0881 (C_10_H_10_N_2_S)	190.0876 (2.63)
				288.1306 (C_16_H_18_N_2_O_2_S)	288.1311 (−1.73)
DP6	Photolytic	C_18_H_23_N_2_O_3_S	347.1125		347.1121 (1.15)
				56.0674 (C_3_H_6_N)	56.0678 (−7.13)
				70.1192 (C_4_H_8_N)	70.1197 (−7.13)
				84.3469 (C_4_H_8_N_2_)	84.3473 (−4.74)
				106.0865 (C_7_H_8_N)	106.0868 (−2.83)
				120.3363 (C_8_H_10_N)	120.3357 (4.99)
				134.0822 (C_7_H_4_NS)	134.0828 (−4.47)
				149.1328 (C_8_H_5_NS)	149.1317 (7.38)
				164.1592 (C_8_H_6_NOS)	164.1597 (−3.05)
				205.0757 (C_10_H_9_N_2_OS)	205.0763 (−2.93)
				223.0854 (C_10_H_11_N_2_O_2_S)	223.0862 (−3.59)
				304.1010 (C_16_H_18_NO_3_S)	304.1015 (−1.64)
DP7	Oxidative	C_18_H_23_N_2_OS	315.1527		315.1517 (3.17)
				74.0845 (C_4_H_12_N)	74.0841 (5.40)
				106.1101 (C_7_H_8_N)	106.1108 (−6.60)
				120.1047 (C_8_H_10_N)	120.1053 (−5.00)
				136.0417 (C_7_H_6_NS)	136.0422 (−3.67)
				191.0902 (C_10_H_11_N_2_S)	191.0907 (−2.62)
				256.2938 (C_16_H_18_NS)	256.2942 (−1.56)

**Table 9:** ADMET and physicochemical properties of the DPs.

Compound	Num. Aromatic Heavy Atoms	Num. Rotatable Bonds	Num. H-Bond Acceptors	Num. H-Bond Donors	TPSA	XLOGP 3	Log S (Ali)	Ali Class	CYP1A2 Inhibitor	CYP2C19 Inhibitor	CYP2C9 Inhibitor	CYP2D6 Inhibitor	CYP3A4 Inhibitor
DP1	6	1	2	2	35.50 Å^2^	1.15	−1.49	Very soluble	No	No	No	No	No
DP2	6	1	1	1	15.27 Å^2^	1.11	−1.02	Very soluble	No	No	No	No	No
DP3	12	3	2	1	51.55 Å^2^	2.90	−3.64	Soluble	No	Yes	No	Yes	Yes
DP4	12	3	4	2	78.02 Å^2^	2.56	−3.85	Soluble	No	No	No	Yes	Yes
DP5	12	3	3	1	57.79 Å^2^	2.91	−3.78	Soluble	No	Yes	Yes	Yes	Yes
DP6	12	3	4	2	78.02 Å^2^	2.56	−3.85	Soluble	No	No	No	Yes	Yes
DP7	12	4	2	2	60.80 Å^2^	2.94	−3.88	Soluble	Yes	No	No	Yes	Yes

## Data Availability

Data are contained within the article and Supplementary Materials.

## Supplementary Materials

The following are available online at https://www.mdpi.com/article/10.3390/molecules27061883/s1, Table S1. Lazar toxicity predictions (https://lazar.in-silico.ch/predict, accessed on 28 November 2021). Figure S1. Q-TOF MS/MS spectra of VOR and its degradation product DP1-DP7. Figure S2. Fragmentation pattern of the [M + H]^+^ ions of VOR and its degradation product DP1-DP7.
