# Supplementary material for: Development of the Validated Stability-Indicating Method for the Determination of Vortioxetine in Bulk and Pharmaceutical Formulation by HPLC-DAD, Stress Degradation Kinetics Studies and Detection of Degradation Products by LC-ESI-QTOF-MS
Source: Molecules. 2022 Mar 14;27(6):1883. doi: 10.3390/molecules27061883 (PMC8953571; doi:10.3390/molecules27061883)
Supplement: Supplementary file 1 [file molecules-27-01883-s001.zip › molecules-1609514-supplementary.pdf]

Article

Development of Validated Stability-Indicating Method for Determination of Vortioxetine in Bulk and Pharmaceutical Formulation by Hplc-Dad, Stress Degradation Kinetics Studies and Detection of Degradation Products by LC-ESI-QTOF-MS

Karol Wróblewski[1,2,3*], Małgorzata Szultka-Młyńska[4], Daria Janiszewska[4], Anna Petruczynik[5], Bogusław Buszewski[4,6]

[1]Department of Experimental and Clinical Pharmacology, University of Rzeszów, Kopisto 2a, 35-959 Rzeszow, Poland; karolw222@wp.pl

[2]Laboratory for Innovative Research in Pharmacology, University of Rzeszów, Kopisto 2a, 35-959 Rzeszow, Poland

[3]Interdisciplinary Center for Preclinical and Clinical Research, University of Rzeszów, Werynia 2A, 36-100 Kolbuszowa, Poland

[4]Department of Environmental Chemistry and Bioanalytics, Nicolaus Copernicus University, Faculty of Chemistry Gagarina 7, 87-100 Torun, Poland; szultka.malgorzata@wp.pl, janiszewska_daria@doktorant.umk.pl

[5]Department of Inorganic Chemistry, Medical University of Lublin, Chodźki 4a, 20-093 Lublin, Poland; annapetruczynik@poczta.onet.pl

[6]Centre for Modern Interdisciplinary Technologies, Nicolaus Copernicus University, Wileńska 4, 87-100 Torun, Poland; bbusz@chem.umk.pl

* Correspondence: karolw222@wp.pl

Supplementary Material

Table S1. Lazar toxicity predictions (https://lazar.in-silico.ch/predict).

Figure S1. Q-TOF MS/MS spectra of VOR and its degradation product DP1-DP7.

Figure S2. Fragmentation pattern of the [M+H]+ ions of VOR and its degradation product DP1-DP7.

Table S1. Lazar toxicity predictions (https://lazar.in-silico.ch/predict).

Copyright: © 2022 by the authors. Licensee MDPI, Basel, Switzerland. This article is an open access article distributed under the terms and conditions of the Creative Commons Attribution (CC BY) license (https://creativecommons.org/licenses/by/4.0/).

Compound		***Acute toxicity (Daphnia magna)***	***Carcinogenicity (Mouse)***	***Carcinogenicity (Rat)***	***Carcinogenicity (Rodents)***	***Mutagenicity (Salmonella typhimurium)***
**DP1**	*Type*	regression	classification	classification	classification	classification
	Prediction	14.8 mg/L	non-carcinogenic	non-carcinogenic	non-carcinogenic	non-mutagenic
	95% Prediction interval	1.56 -142 mg/L	NA	NA	NA	NA

	Probability	NA	non-carcinogenic: 0.207<br>carcinogenic: 0.066	carcinogenic: 0.13<br>non-carcinogenic: 0.143	carcinogenic: 0.06<br>non-carcinogenic: 0.213	mutagenic: 0.255<br>non-mutagenic: 0.29
**DP2**	*Type*	regression	classification	classification	classification	classification
	Prediction	2.36 mg/L	non-carcinogenic	**carcinogenic**	non-carcinogenic	mutagenic;<br>attention:<br>measured activity:<br>non-mutagenic
	95% Prediction interval	0.111 - 50.5 mg/L	NA	NA	NA	NA
	Probability	NA	non-carcinogenic: 0.244<br>carcinogenic: 0.0892	carcinogenic: 0.193<br>non-carcinogenic: 0.141	carcinogenic: 0.0989<br>non-carcinogenic: 0.136	mutagenic: 0.254<br>non-mutagenic: 0.191
**DP3**	*Type*	regression	classification	classification	classification	classification
	Prediction	17.5 mg/L	non-carcinogenic	**carcinogenic**	**carcinogenic**	**mutagenic**
	95% Prediction interval	0.172 – 1780.0 mg/L	NA	NA	NA	NA
	Probability	NA	non-carcinogenic: 0.153<br>carcinogenic: 0.141	carcinogenic: 0.191<br>non-carcinogenic: 0.103	carcinogenic: 0.246<br>non-carcinogenic: 0.0481	mutagenic: 0.184<br>non-mutagenic: 0.15
**DP4**	*Type*	regression	classification	classification	classification	classification
	Prediction	47.1 mg/L	non-carcinogenic	**carcinogenic**	**carcinogenic**	non-mutagenic

	95% Prediction interval	3.12 – 713.0 mg/L	NA	NA	NA	NA
	Probability	NA	non-carcinogenic: 0.263<br>carcinogenic: 0.0	carcinogenic: 0.222<br>non-carcinogenic: 0.0415	carcinogenic: 0.263<br>non-carcinogenic: 0.0	mutagenic: 0.0709<br>non-mutagenic: 0.215
**DP5**	*Type*	regression	classification	classification	classification	classification
	Prediction	17.3 mg/L	non-carcinogenic	**carcinogenic**	**carcinogenic**	**mutagenic**
	95% Prediction interval	0.24 -1240.0 mg/L	NA	NA	NA	NA
	Probability	NA	non-carcinogenic: 0.17<br>carcinogenic: 0.124	carcinogenic: 0.21<br>non-carcinogenic: 0.084	carcinogenic: 0.253<br>non-carcinogenic: 0.0413	mutagenic: 0.171<br>non-mutagenic: 0.162
**DP6**	*Type*	regression	Warnings:<br>cannot create pre-diction: only one similar compound for threshold 0.2 in the training set (Threshold: 0.2).	classification	Warnings:<br>cannot create pre-diction: only one similar compound for threshold 0.2 in the training set (Threshold: 0.2).	classification
	Prediction	34.6 mg/L		non-carcinogenic		non-mutagenic
	95% Prediction interval	3.26 -367.0 mg/L		NA		NA
	Probability	NA		carcinogenic: 0.0829<br>non-carcinogenic: 0.167		mutagenic: 0.132<br>non-mutagenic: 0.168

**DP7**	*Type*	regression	classification	classification	classification	classification
	Prediction	45.4 mg/L	non-carcinogenic	**carcinogenic**	**carcinogenic**	non-mutagenic
	95% Prediction interval	Warnings:<br><br>Insufficient number of neighbors (2) for regression model, using weighted average of similar substances (no prediction interval available).	NA	NA	NA	NA
	Probability	NA	non-carcinogenic: 0.217<br>carcinogenic: 0.0611	carcinogenic: 0.167<br>non-carcinogenic: 0.11	carcinogenic: 0.151<br>non-carcinogenic: 0.127	mutagenic: 0.0628<br>non-mutagenic: 0.253

NA-not applicable

(a)

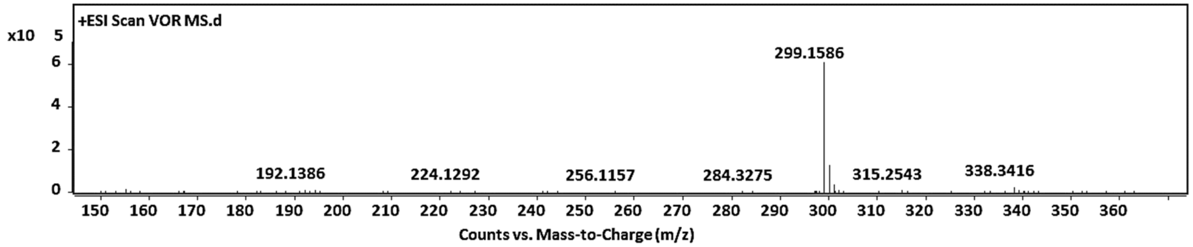

(b)

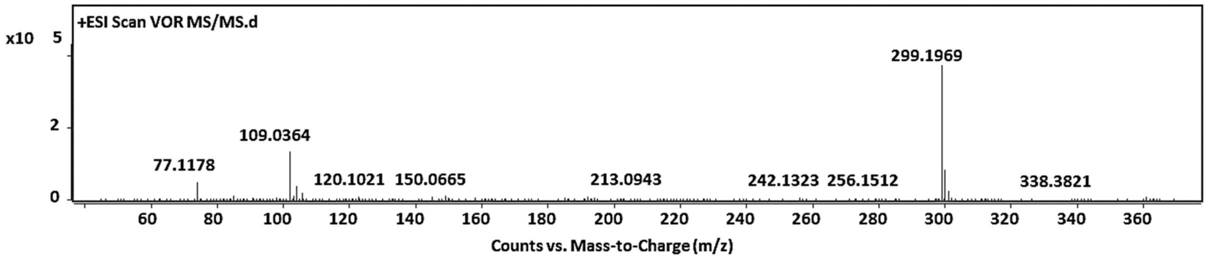
+ESI Scan VOR MS/MS.d
x10 5
299.1969
109.0364
77.1178
120.1021
150.0665
213.0943
242.1323
256.1512
338.3821
Counts vs. Mass-to-Charge (m/z)

(c)

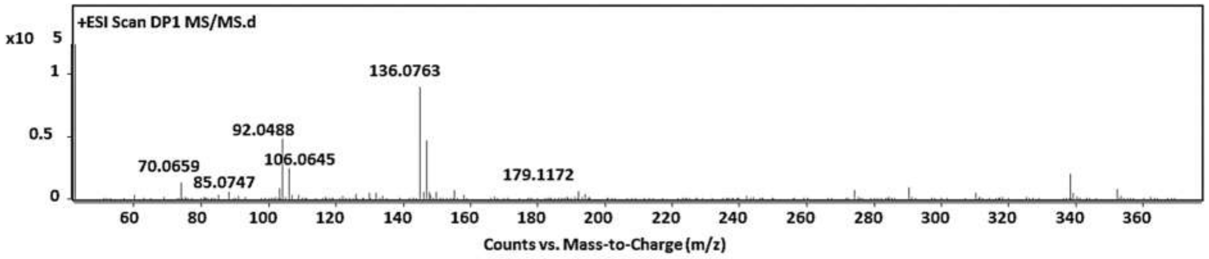
+ESI Scan DP1 MS/MS.d
x10 5
136.0763
92.0488
106.0645
70.0659
85.0747
179.1172
Counts vs. Mass-to-Charge (m/z)

(d)

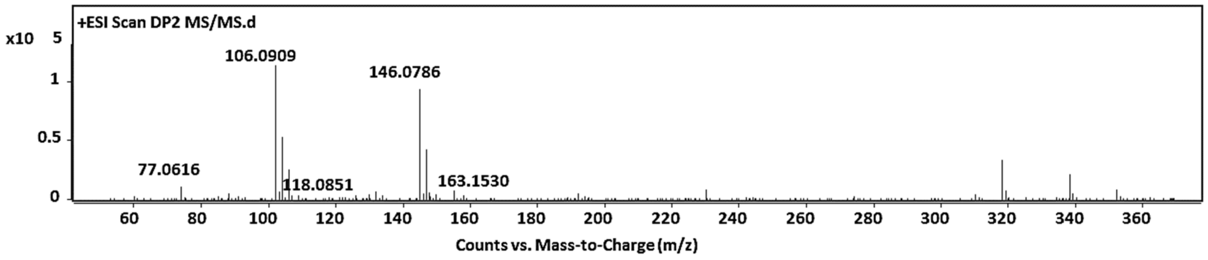
+ESI Scan DP2 MS/MS.d
x10 5
106.0909
146.0786
77.0616
118.0851
163.1530
Counts vs. Mass-to-Charge (m/z)

(e)

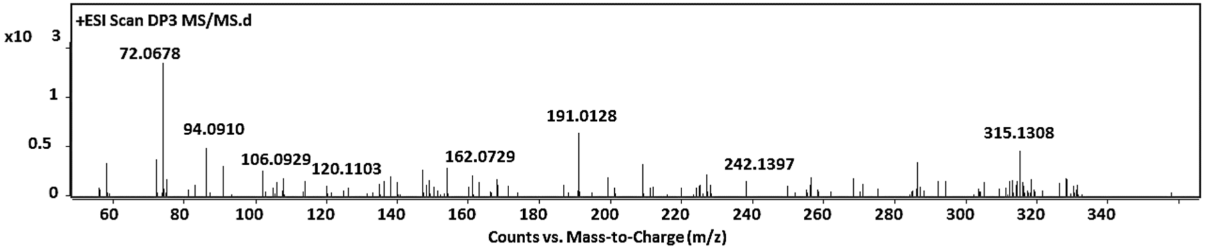
+ESI Scan DP3 MS/MS.d
x10 3
72.0678
94.0910
106.0929
120.1103
162.0729
191.0128
242.1397
315.1308
Counts vs. Mass-to-Charge (m/z)

(f)

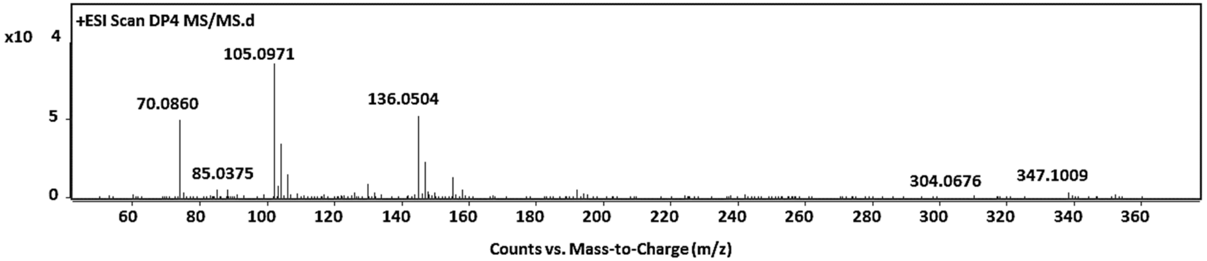
+ESI Scan DP4 MS/MS.d
x10 4
105.0971
70.0860
136.0504
85.0375
304.0676
347.1009
Counts vs. Mass-to-Charge (m/z)

(g)

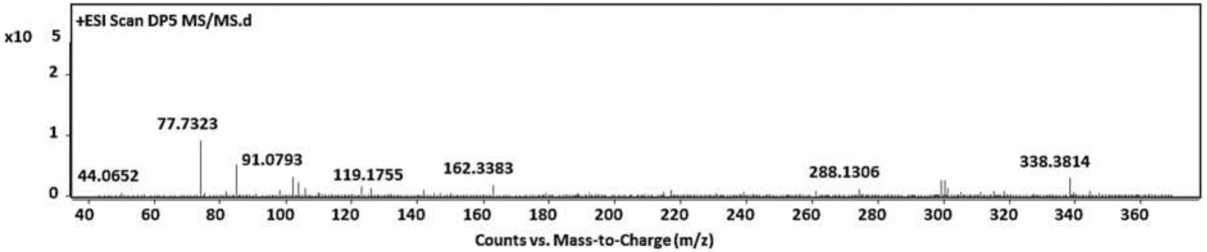
+ESI Scan DP5 MS/MS.d
x10 5
77.7323
44.0652
91.0793
119.1755
162.3383
288.1306
338.3814
Counts vs. Mass-to-Charge (m/z)

(h)

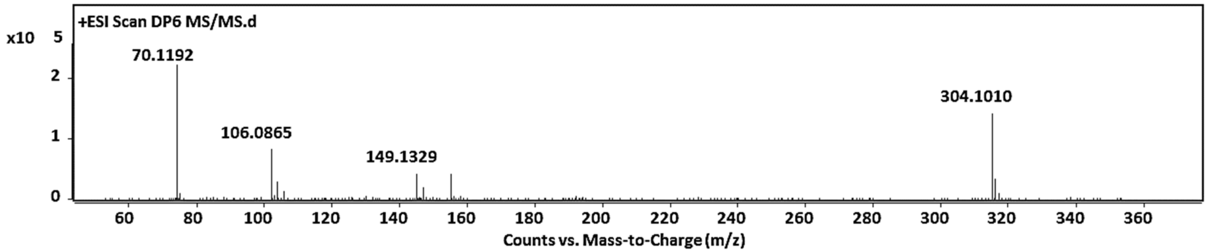

(i)

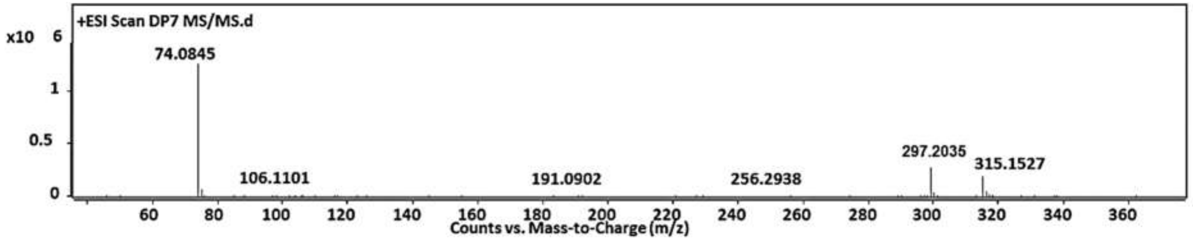

Figure S1. Q-TOF MS/MS spectra of VOR and its degradation product DP1-DP7.

(a)

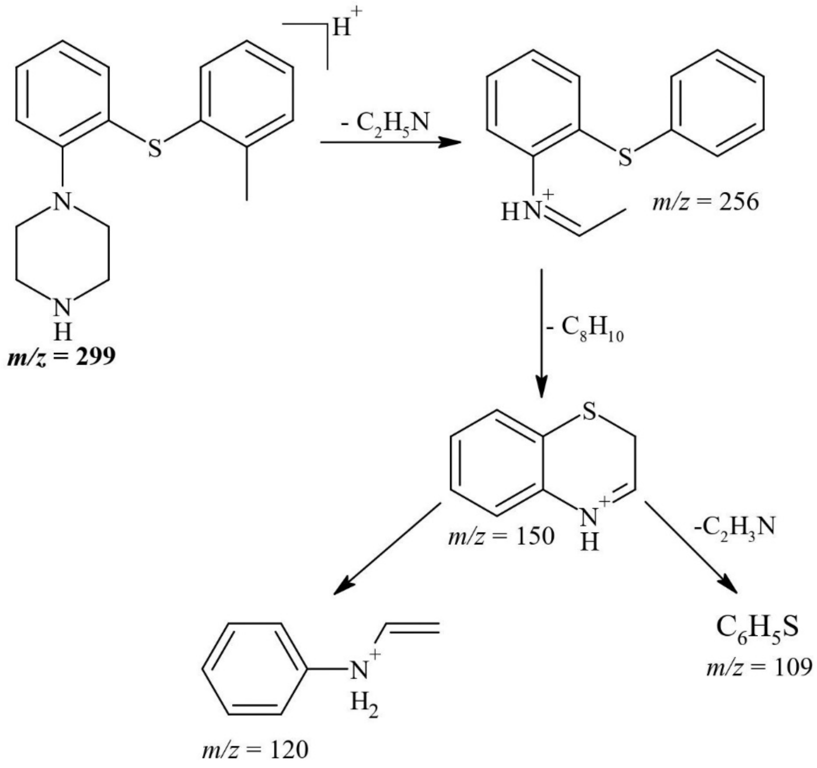

(b)

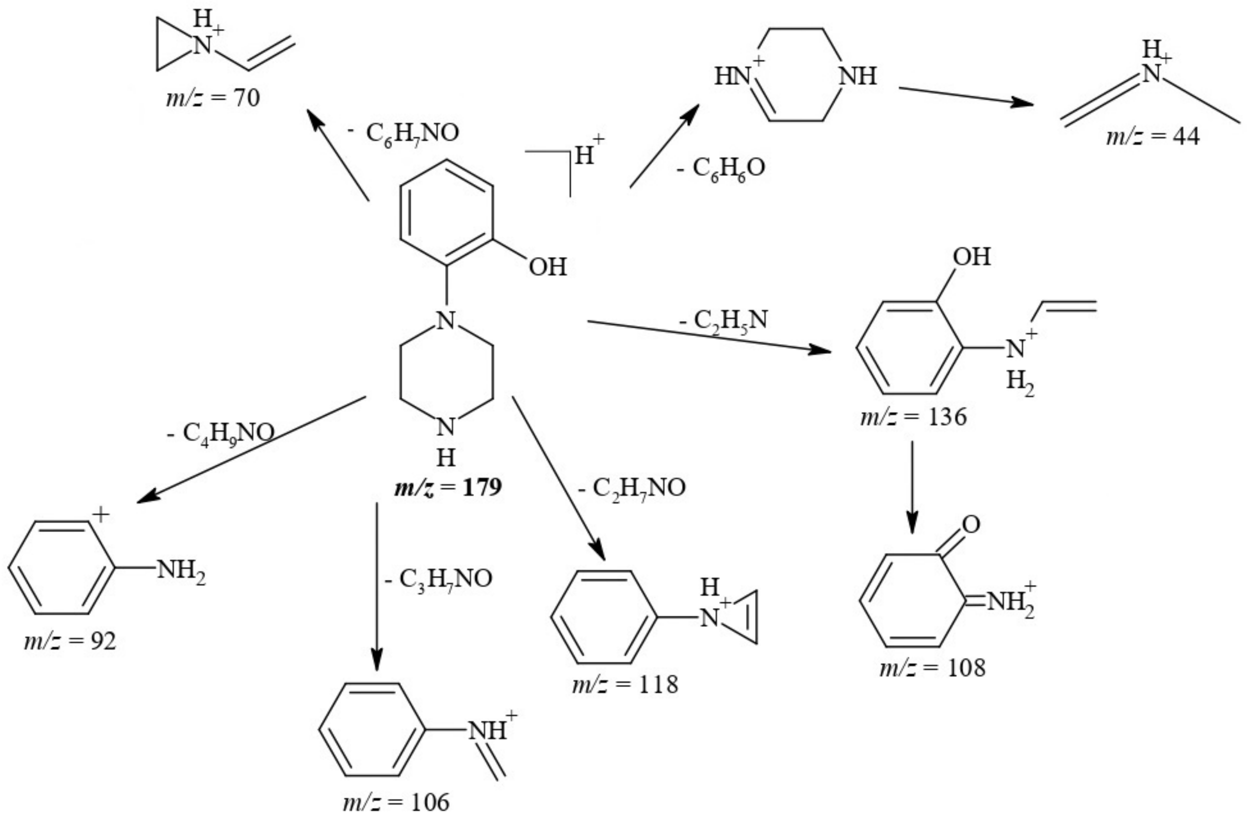
m/z = 70
- C6H7NO
H+
- C6H6O
HN+
NH
m/z = 44
OH
N
N
H
m/z = 179
- C2H5N
OH
N+
H2
m/z = 136
- C4H9NO
NH2
m/z = 92
- C3H7NO
m/z = 106
NH+
- C2H7NO
m/z = 118
O
NH2+
m/z = 108

(c)

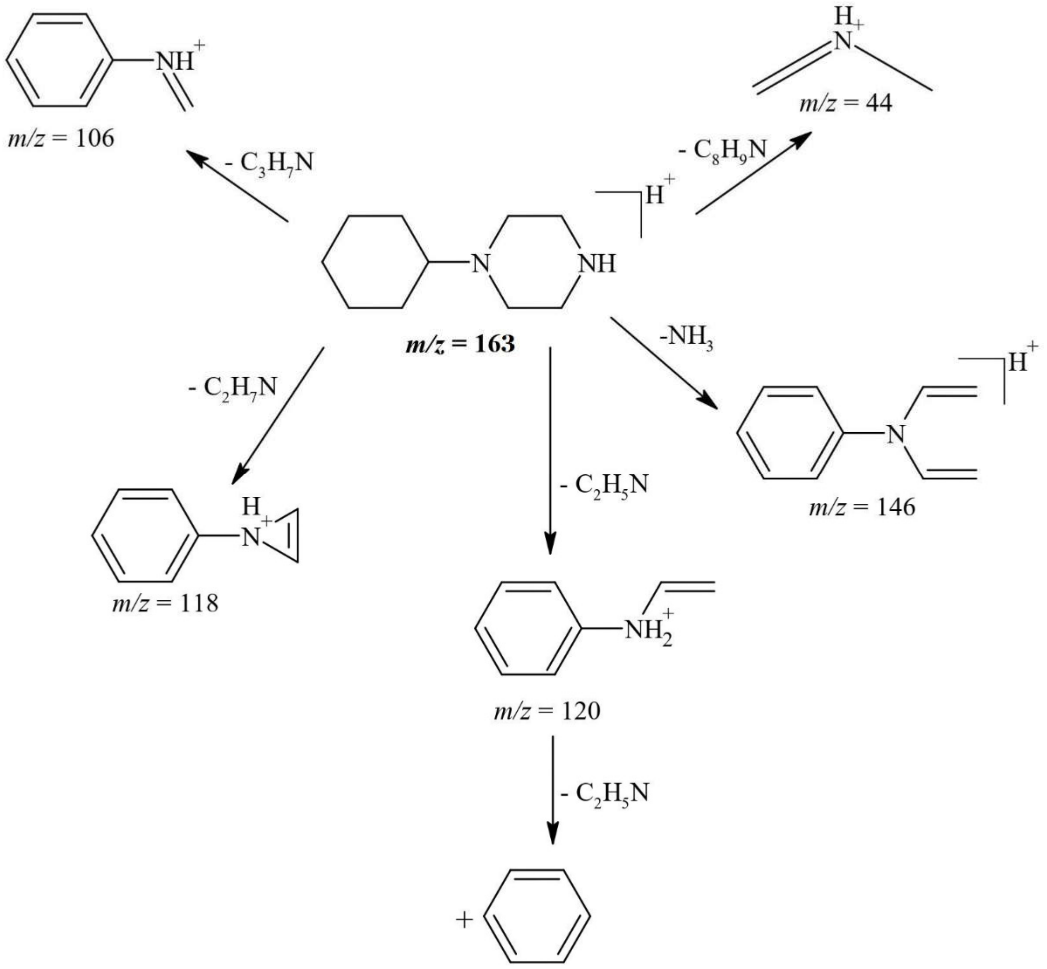
m/z = 106
- C3H7N
- C8H9N
m/z = 44
H+
m/z = 163
-NH3
- C2H7N
- C2H5N
m/z = 146
m/z = 118
m/z = 120
- C2H5N
+

(d)

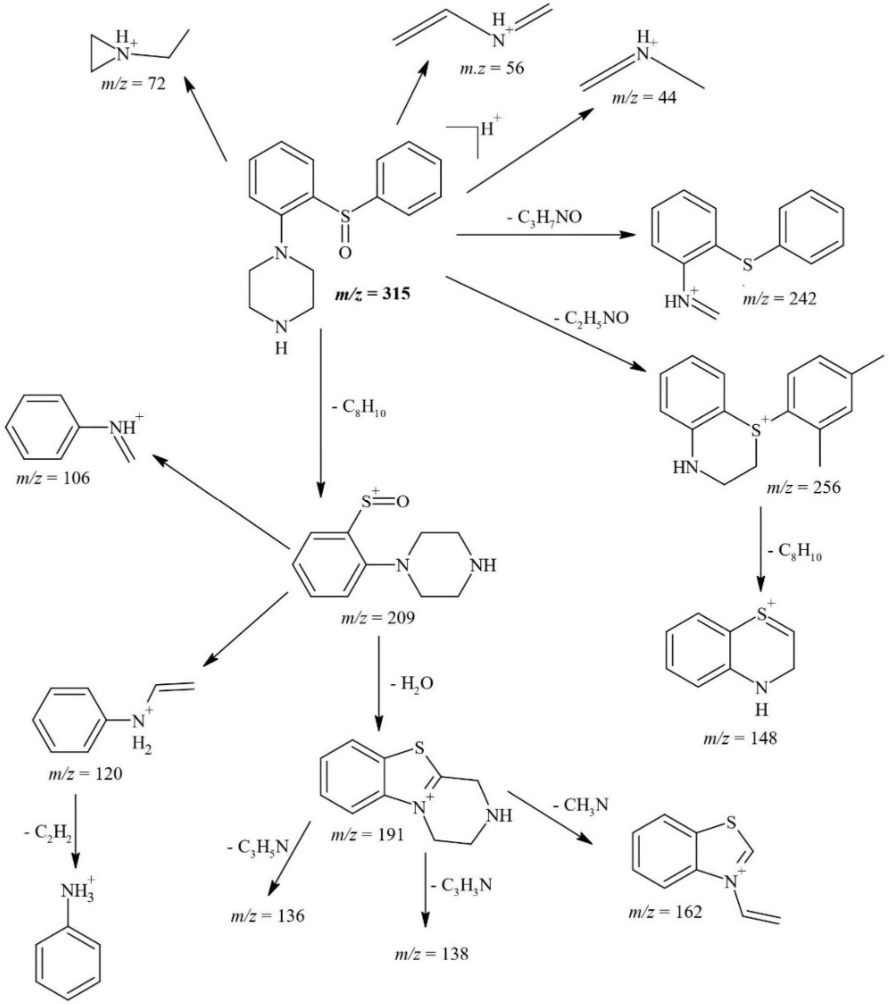
m/z = 72
m.z = 56
m/z = 44
m/z = 315
- C3H7NO
m/z = 242
- C2H3NO
m/z = 256
- C8H10
m/z = 106
m/z = 209
- C8H10
m/z = 148
- H2O
m/z = 120
- C2H2
m/z = 191
- CH3N
- C3H5N
- C3H3N
m/z = 136
m/z = 138
m/z = 162

(e)

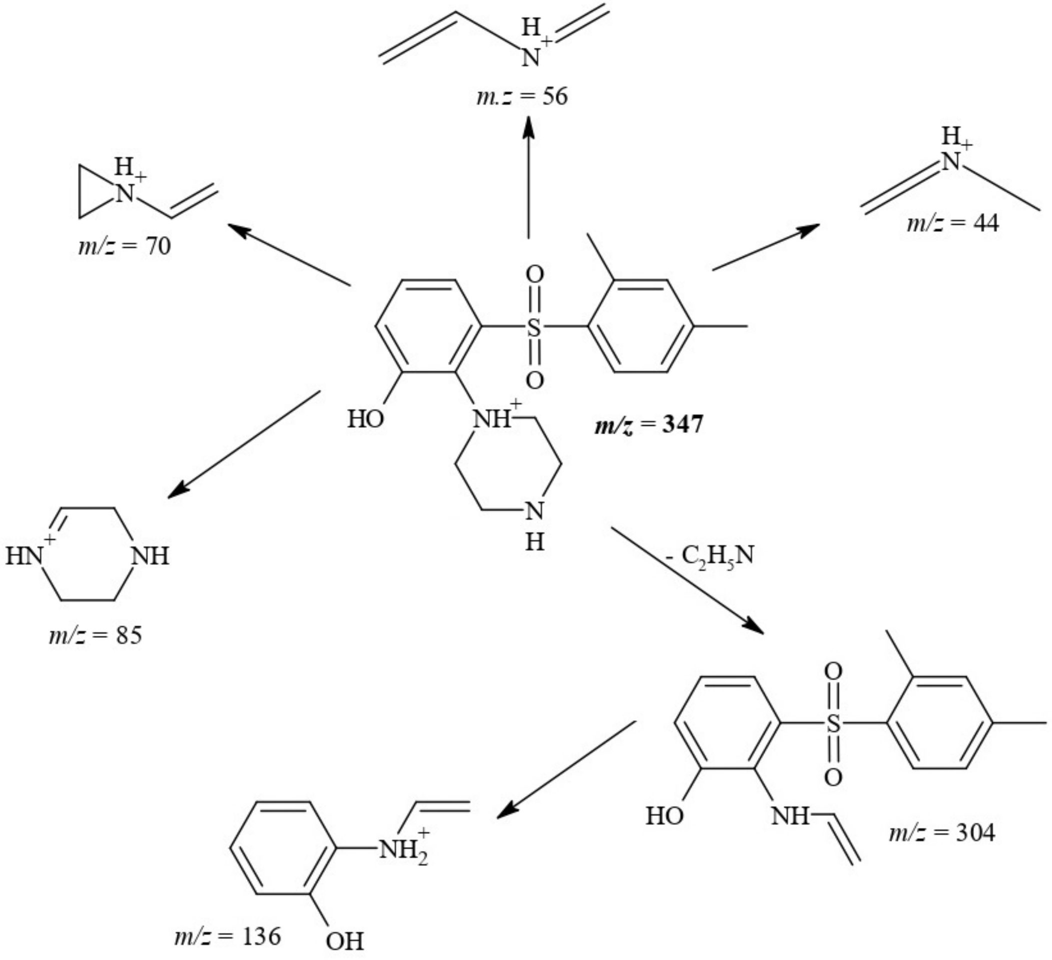

(f)

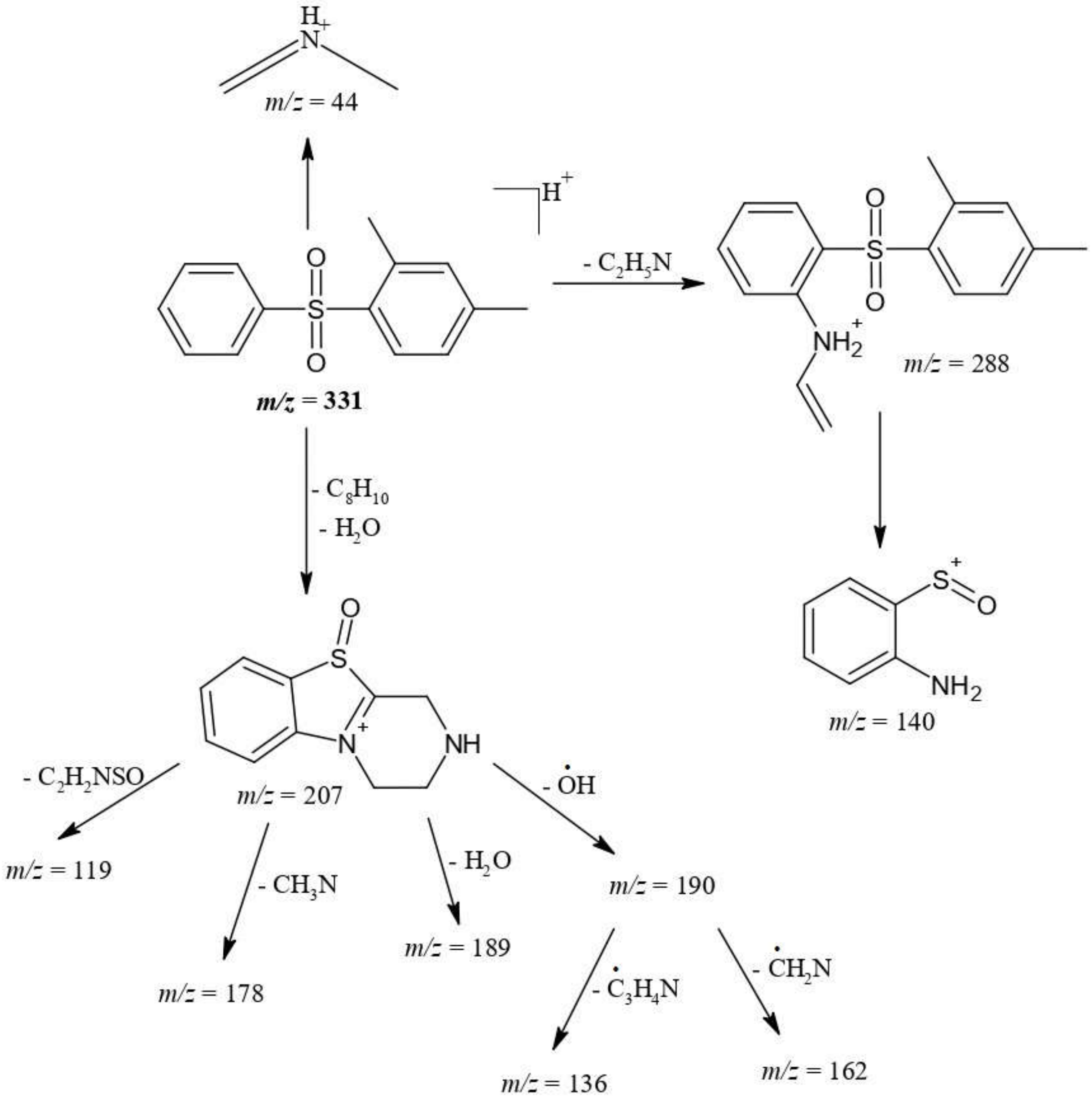

m/z = 44
m/z = 331
- C2H5N
m/z = 288
- C8H10
- H2O
m/z = 140
m/z = 207
- C2H2NSO
m/z = 119
- CH3N
m/z = 178
- H2O
m/z = 189
- OH
m/z = 190
- C3H4N
m/z = 136
- CH2N
m/z = 162

(g)

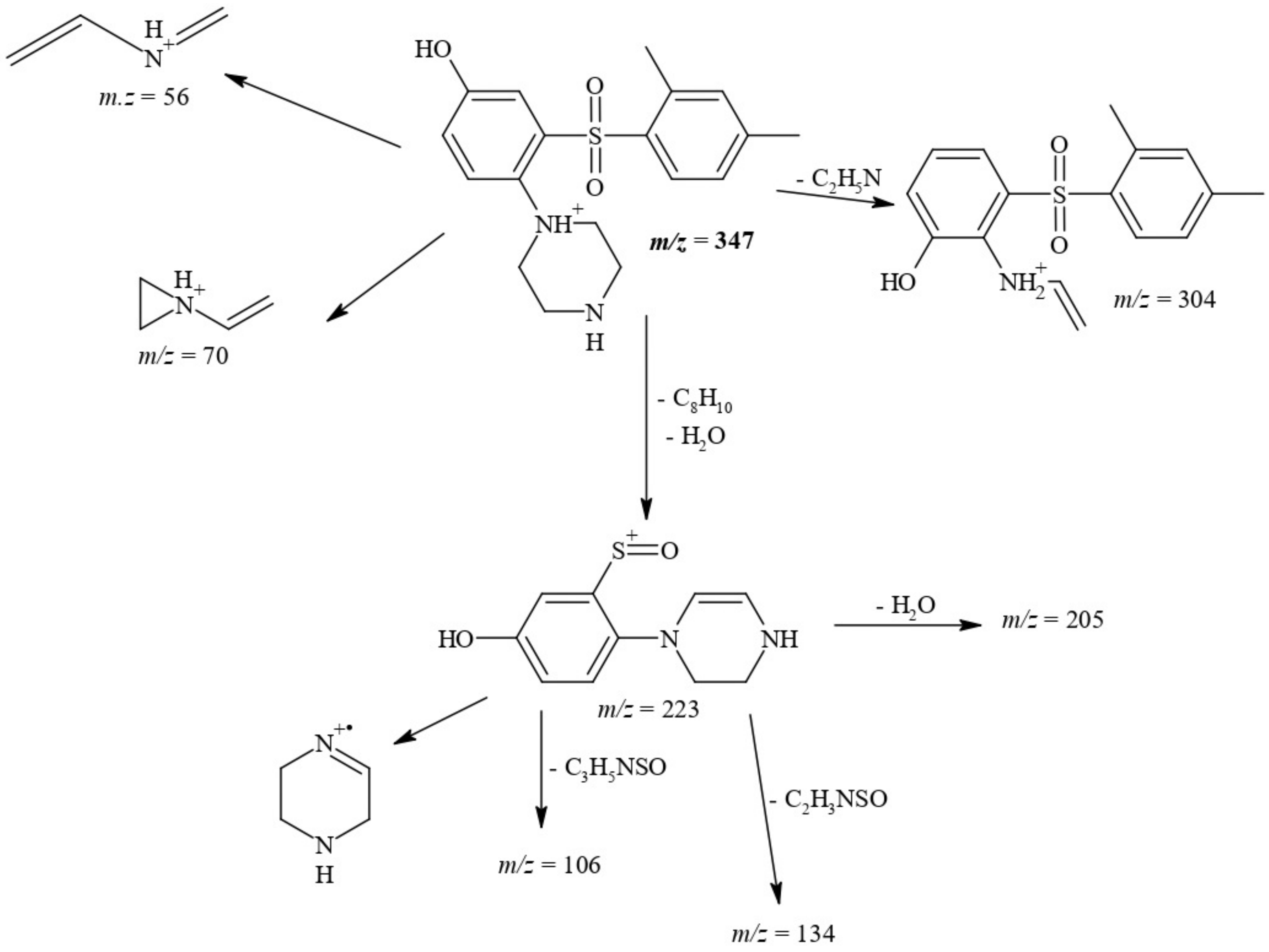
HO
O
S
O
NH+
N
H
m/z = 347
m.z = 56
m/z = 70
- C2H5N
HO
NH2+
m/z = 304
- C8H10
- H2O
S+=O
HO
N
NH
m/z = 223
- H2O
m/z = 205
N+•
N
H
- C3H5NSO
m/z = 106
- C2H3NSO
m/z = 134

(h)

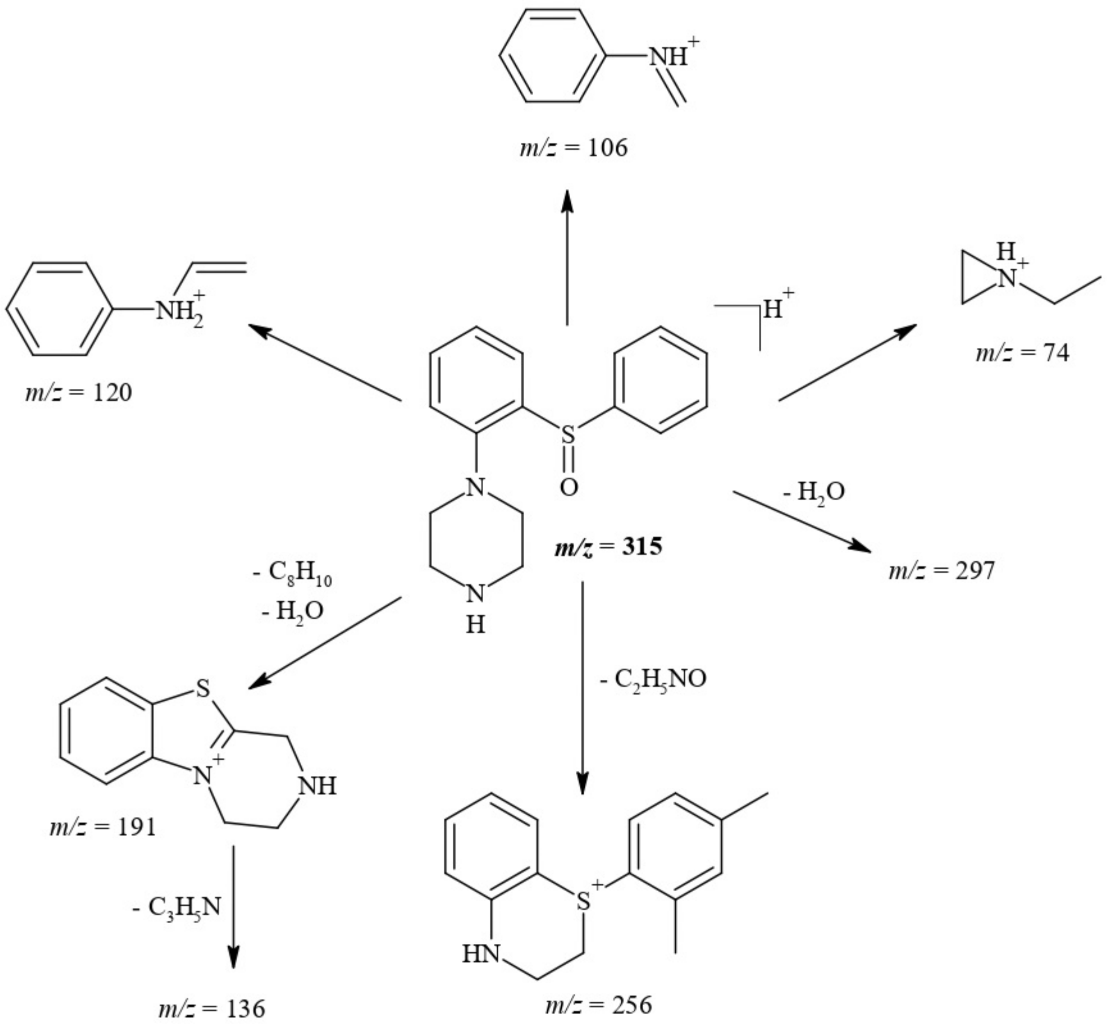

Figure S2. Fragmentation pattern of the [M+H]+ ions of VOR and its degradation product DP1-DP7.
